# Twa1/Gid8 is a β-catenin nuclear retention factor in Wnt signaling and colorectal tumorigenesis

**DOI:** 10.1038/cr.2017.107

**Published:** 2017-08-22

**Authors:** Yi Lu, Shanshan Xie, Wen Zhang, Cheng Zhang, Cheng Gao, Qiang Sun, Yuqi Cai, Zhangqi Xu, Min Xiao, Yanjun Xu, Xiao Huang, Ximei Wu, Wei Liu, Fudi Wang, Yibin Kang, Tianhua Zhou

**Affiliations:** 1 Department of Cell Biology and Program in Molecular Cell Biology, Zhejiang University School of Medicine, Hangzhou, Zhejiang 310058, China; 2 Collaborative Innovation Center for Diagnosis and Treatment of Infectious Diseases, Hangzhou, Zhejiang 310003, China; 3 Zhejiang Cancer Hospital, Hangzhou, Zhejiang 310022, China; 4 Institute of Cellular and Developmental Biology, College of Life Sciences, Zhejiang University, Hangzhou, Zhejiang 310058, China; 5 Department of Molecular Biology, Princeton University, Princeton, NJ 08540, USA; 6 Current address: Division of Pulmonary Biology, Cincinnati Children's Hospital, Cincinnati, OH 45229, USA

**Keywords:** Twa1, β-catenin nuclear retention, Wnt signaling, zebrafish development, colorectal tumorigenesis

## Abstract

Hyperactivation of Wnt/β-catenin signaling is one of the major causes of human colorectal cancer (CRC). A hallmark of Wnt signaling is the nuclear accumulation of β-catenin. Although β-catenin nuclear import and export have been widely investigated, the underlying mechanism of β-catenin's nuclear retention remains largely unknown. Here, we report that Twa1/Gid8 is a key nuclear retention factor for β-catenin during Wnt signaling and colorectal carcinogenesis. In the absence of Wnt, Twa1 exists together with β-catenin in the Axin complex and undergoes ubiquitination and degradation. Upon Wnt signaling, Twa1 translocates into the nucleus, where it binds and retains β-catenin. Depletion of Twa1 attenuates Wnt-stimulated gene expression, dorsal development of zebrafish embryos and xenograft tumor growth of CRC cells. Moreover, nuclear Twa1 is significantly upregulated in human CRC tissues, correlating with the nuclear accumulation of β-catenin and poor prognosis. Thus, our results identify Twa1 as a previously undescribed regulator of the Wnt pathway for promoting colorectal tumorigenesis by facilitating β-catenin nuclear retention.

## Introduction

Canonical Wnt signaling plays pivotal roles in many biological processes, such as cell proliferation, cell fate determination and stem cell maintenance^1,2^. Dysfunction of the Wnt cascade is implicated in a variety of human diseases, most prominently in colorectal cancer (CRC). Mutation in the *adenomatous polyposis coli* (*APC*) gene, a key component of this pathway, is thought to be an initiating event in human colorectal carcinogenesis. In mouse models, inactivation of the *APC* gene causes intestinal hyperplasia and adenocarcinoma^3^.

A hallmark of canonical Wnt signaling is the stabilization and nuclear translocation of β-catenin^4,5,6^. In the absence of Wnt, cytoplasmic β-catenin is constitutively degraded by the Axin complex, which is composed of APC, Axin, casein kinase 1 (CK1) and glycogen synthase kinase 3 (GSK3). CK1 and GSK3 sequentially phosphorylate β-catenin, resulting in its ubiquitination and proteasomal degradation. Upon Wnt stimulation, the Wnt ligand triggers the inactivation of the Axin complex to stabilize β-catenin, which subsequently translocates into the nucleus to form a transcriptional complex with T-cell factor (TCF) to activate Wnt target gene expression. Previous studies have reported that some proteins, including forkhead box protein M1 (FoxM1), mucin-1, insulin receptor substrate-1 (IRS-1) and B-cell lymphoma 9 (BCL9), facilitate β-catenin import into the nucleus, whereas other proteins, such as APC, Axin, Ran binding protein 3 (RanBP3) and Chibby, actively promote β-catenin export out of the nucleus^7,8,9,10,11,12,13,14,15,16^. These findings indicate that the nuclear β-catenin level is fine-tuned at multiple layers, such as protein stability and nuclear import and export. However, little is known about whether and how nuclear retention might provide another layer of regulation to control β-catenin nuclear accumulation.

Here we show that Twa1 (two hybrid-associated protein no.1 with RanBPM), also known as Gid8 (glucose-induced degradation protein 8 homolog), is a key nuclear retention factor for β-catenin during Wnt signaling and colorectal tumorigenesis. Twa1/Gid8 (Twa1) was initially described in the hunt for the proteins that can interact with Ran binding protein M (RanBPM), but its biological function remains largely unclear^17,18^. Using a comprehensive approach, we find that Twa1 is not only significantly upregulated in human CRC tissues, but also that this correlates with a poor prognosis for CRC patients. In response to Wnt signaling, Twa1 directly binds to β-catenin and promotes its nuclear retention. We exemplify the functions of Twa1 by showing its requirement for the dorsal development of zebrafish embryos and for the growth of CRC cells in a xenograft model. Although Twa1 associates with β-catenin in the nucleus, it does not interact with either TCF4 or Wnt-responsive elements (WREs), suggesting a chromatin-independent retention mechanism that is indispensible for the Wnt transcriptional program.

## Results

### *Twa1* is upregulated in human CRC tissues

**Figure 1:**
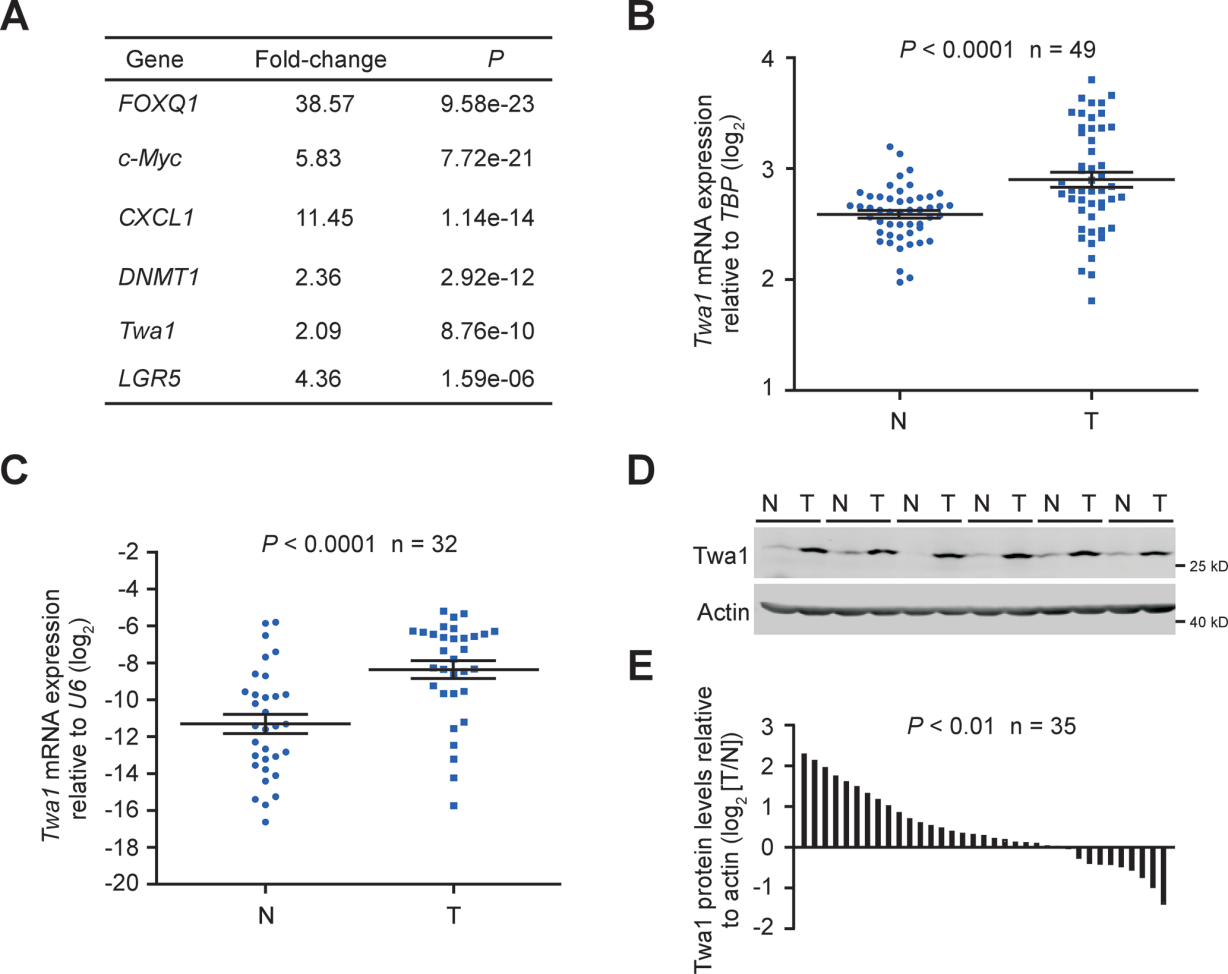
Twa1 is significantly upregulated in human CRC tissues. **(A)** Bioinformatics analysis of *Twa1* expression in human CRC tissues and nontumor tissues from the Hong CRC microarray data set (GSE9348) available in the Oncomine database. Some genes highly significantly associated with CRC are listed. *CXCL1*, chemokine (C-X-C motif) ligand 1; *DNMT1*, DNA (cytosine-5-)-methyltransferase 1; *FOXQ1*, fork-head box Q1; *LGR5*, leucine-rich repeat containing G protein-coupled receptor 5. **(B**, **C)** Relative expression of *Twa1* mRNA in human CRC tissues and their matched nontumor tissues in the CRC RNA-seq data set obtained from the TCGA database **(B)** and in quantitative RT-PCR (qRT-PCR) analysis of our own clinical samples **(C)**. Each point represents log_2_ transformed *Twa1* expression relative to either *TBP* (TATA binding protein) or small nuclear RNA *U6* expression in a single sample. Black horizontal bars show the median ± SD. *P* < 0.0001, Student's *t*-test. **(D**, **E)** Western analysis of Twa1 protein levels in CRC tissues and their matched nontumor tissues. The densities of Twa1 bands are quantified by Image J software (NIH) and normalized to actin, a loading control. Data are presented as log_2_ value of Twa1 (T/N). *P* < 0.01, Student's *t*-test.

To explore the molecular mechanism of colorectal carcinogenesis, we analyzed the global gene expression profile of a cohort of human CRC tissues from the Oncomine database (GSE9348)^19^. The results showed that the expression of 2 549 genes in CRC tissues was significantly changed when compared to nontumor tissues (fold change > 2, *P* < 0.01, false discovery rate (FDR) < 0.01) (Supplementary information, Figure S1A). As expected, there were a number of well-known CRC-associated genes, including *fork-head box Q1* (*FoxQ1*), *c-Myc* and *leucine-rich repeat containing G protein-coupled receptor* *5* (*LGR5*), in our analysis (Figure 1A)^20,21,22^. Among the changed genes, a poorly defined gene, *Twa1*, was significantly upregulated in CRC tissues (*P* = 8.76e-10). To confirm the upregulation of *Twa1* in CRC tissues, we analyzed the RNA-seq data from the TCGA database and found that *Twa1* mRNA expression levels were also significantly higher in CRC tissues than their pair-matched nontumor samples (Figure 1B). Moreover, we validated that both *Twa1* mRNA and protein levels were significantly increased in our own set of CRC samples (Figure 1C-1E). Taken together, these data suggest that Twa1 expression is associated with colorectal tumorigenesis.

### Twa1 is required for nuclear accumulation of β-catenin during Wnt activation

**Figure 2:**
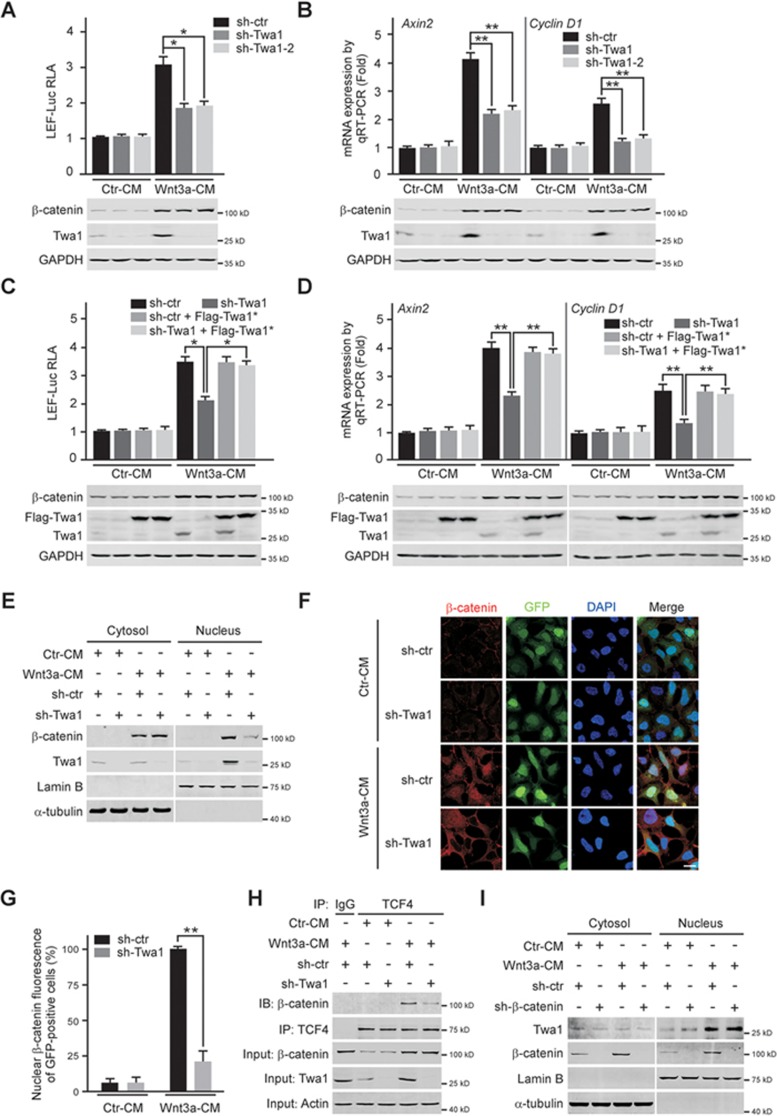
Twa1 promotes β-catenin nuclear accumulation and target gene expression in response to Wnt signaling. HEK-293 cells treated with lentivirus-based shRNAs targeting different regions of *Twa1* mRNA (sh-Twa1 and sh-Twa1-2), *β-catenin* mRNA (sh-β-catenin) or control shRNA (sh-ctr) were transfected with an RNAi-resistant human Twa1 (Twa1^*^) construct or not. The cells were then treated with Wnt3a-conditioned medium (Wnt3a-CM) or control medium (Ctr-CM), and subjected to the following analyses. **(A-D)** Effect of Twa1 depletion on Wnt-induced luciferase reporter activity **(A**, **C)** or Wnt target gene expression **(B**, **D)**. Quantitative data are expressed as the mean ± SEM (at least three independent experiments). ^*^P < 0.05 and ^**^P < 0.01, Student's *t*-test. **(E)** Western analysis of endogenous β-catenin and Twa1 from cytosolic and nuclear fractions of HEK-293 cells. Lamin B and α-tubulin were used as loading controls for nuclear and cytoplasmic fractions, respectively. **(F**, **G)** Confocal microscopy of β-catenin nuclear localization. Green signals represent cells infected with lentiviruses. DNA was visualized with DAPI (blue). The intensity of red signals in the nuclei of GFP-positive cells was plotted. The nuclear intensity in the GFP-positive cells treated with both sh-ctr and Wnt3a-CM is set as 100%. More than 50 nuclei are measured in each group. Scale bars, 10 μm. ^**^P < 0.01, Student's *t*-test. **(H)** Co-IP analysis with the indicated antibodies showing the interaction between endogenous β-catenin and TCF4. **(I)** Western analysis with the indicated antibodies showing the effect of β-catenin depletion on Twa1 nuclear accumulation upon Wnt3a stimulation. IB, immunoblotting; IP, immunoprecipitation.

To determine the regulation of Twa1 in colorectal carcinogenesis, we first examined several important signaling pathways involved in CRC by dual luciferase reporter assays and observed that Twa1 depletion selectively impaired Wnt signaling in HEK-293 cells (Supplementary information, Figures S2 and S3)^23,24,25,26,27,28^. Further analyses showed that knockdown of *Twa1* by two shRNAs targeting different regions of *Twa1* mRNA significantly inhibited lymphoid enhancer-binding factor-luciferase (LEF-Luc) activity and reduced the mRNA levels of Wnt target genes, *Axin2* and *Cyclin D1* (Figure 2A and 2B). These effects were efficiently reversed by ectopic expression of RNAi-resistant Twa1 (Figure 2C and 2D). Thus, these results imply that Twa1 plays an important role in canonical Wnt signaling.

To investigate the role of Twa1 in the Wnt pathway, we continued to examine the β-catenin protein level in Twa1-depleted HEK-293 cells. In response to Wnt3a, *Twa1* knockdown did not obviously change the levels of Wnt-induced β-catenin (Figure 2A-2D), but substantially reduced the levels of nuclear β-catenin (Figure 2E), which was confirmed by immunostaining (Figure 2F and 2G). Similar results were also observed in RKO cells that are commonly used for Wnt signaling studies (Supplementary information, Figure S4). Further co-immunoprecipitation (co-IP) experiments verified that the interaction between β-catenin and the transcription factor TCF4 was also decreased in Twa1-depleted cells (Figure 2H). In addition, silencing of Twa1 considerably suppressed β-catenin nuclear accumulation and Wnt target gene expression induced by the GSK3β inhibitor lithium chloride (LiCl) (Supplementary information, Figure S5). Interestingly, depletion of β-catenin had no apparent effect on nuclear Twa1 levels (Figure 2I), suggesting that Twa1 is critical for β-catenin nuclear accumulation, but not *vice versa*.

To further validate the function of Twa1 in β-catenin nuclear accumulation, we generated *Twa1* knockout (KO) HEK-293 cell lines using the CRISPR/Cas9 (clustered regularly interspaced short palindromic repeats/CRISPR-associated protein 9) system. Two *Twa1* KO cell lines were obtained and the deletion was verified by genomic DNA PCR, Sanger sequencing and western blotting (Supplementary information, Figure S6A-S6C). Deletion of *Twa1* gene obviously suppressed Wnt-induced LEF-Luc reporter activity, Wnt target gene expression, β-catenin nuclear accumulation and the TCF4-β-catenin interaction (Supplementary information, Figure S6D-S6H). Taken together, these data indicate an essential role of Twa1 in β-catenin nuclear accumulation during Wnt activation.

### Twa1 facilitates β-catenin nuclear accumulation through its CRA domain

To explore the mechanism of Twa1-dependent β-catenin nuclear accumulation, we first investigated the association between Twa1 and β-catenin. An *in vitro* GST pull-down assay showed that purified GST-Twa1 directly interacted with His-β-catenin (Supplementary information, Figure S7A). Immunoprecipitation analysis revealed that either endogenous or ectopically expressed Twa1 interacted with endogenous β-catenin (Supplementary information, Figure S7B and S7C). Importantly, Wnt3a treatment not only increased the levels of nuclear Twa1 and β-catenin, but also enhanced their interaction in the nucleus (Supplementary information, Figure S7D).

**Figure 3:**
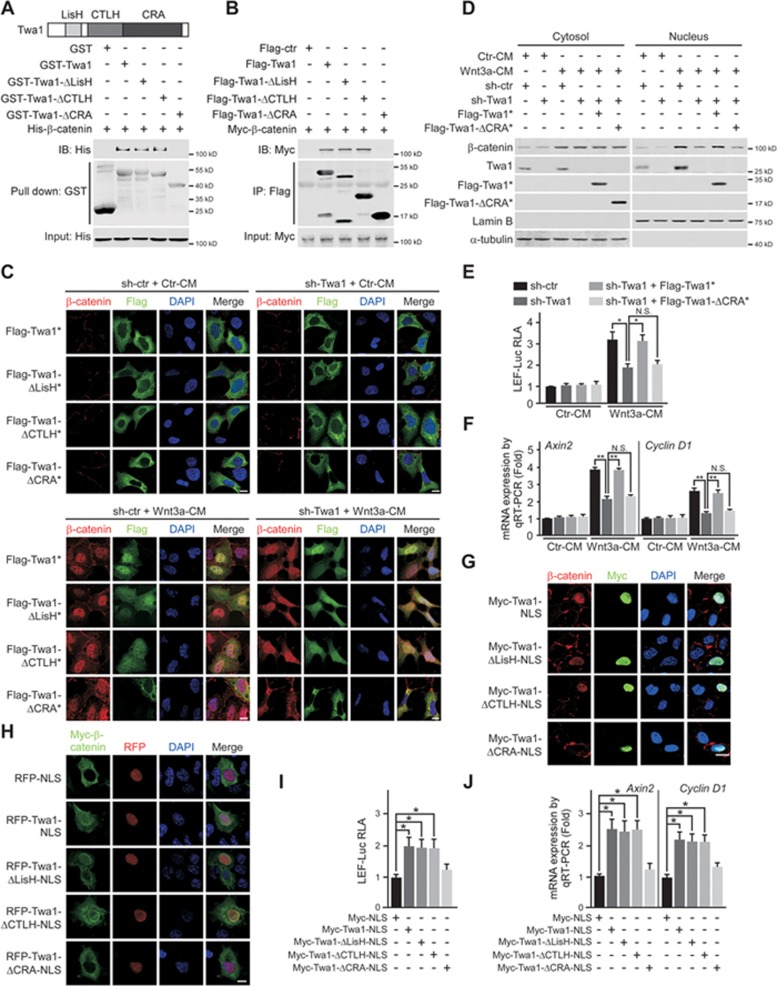
Twa1 facilitates Wnt-induced β-catenin nuclear accumulation through its CRA domain. **(A)** GST pull-down analysis of purified His-β-catenin and wild-type or mutant GST-Twa1 *in vitro*. LisH, Lis1 homology domain; CTLH, C-terminal to LisH domain; CRA, CT11-RanBPM domain. **(B)** HEK-293 cells transfected with the indicated constructs were subjected to co-IP and subsequent western analysis. **(C-F)** HEK-293 cells infected with sh-Twa1- or sh-ctr-containing lentiviruses were transfected with RNAi-resistant wild-type or mutant human Twa1 constructs, treated with Wnt3a-CM or Ctr-CM, and then processed for immunoflouresence **(C)**, western blotting **(D)**, luciferase reporter assays **(E)** and Wnt target gene expression analysis **(F)**. Scale bars, 10 μm. **(G-J)** HEK-293 cells transfected with wild-type or mutant Twa1 constructs containing NLS sequence were subjected to immunoflouresence **(G**, **H)**, luciferase reporter assays **(I)** and Wnt target gene expression analysis **(J)**. DNA was visualized with DAPI (blue). Bars, 10 μm. Quantitative data are expressed as the mean ± SEM (at least three independent experiments). ns, not significant. ^*^P < 0.05 and ^**^P < 0.01, Student's *t*-test.

To delineate the region of Twa1 responsible for its interaction with β-catenin, we performed GST pull-down and co-IP experiments and discovered that the conserved CRA (CT11-RanBPM) domain of Twa1 was required for its interaction with β-catenin (Figure 3A and 3B)^17,18^. To test whether the CRA domain of Twa1 is required for Wnt activation, we ectopically expressed RNAi-resistant wild-type or mutant Twa1 in HEK-293 cells depleted of Twa1. Without Wnt stimulation, wild-type or mutant Twa1 was preferentially localized in the cytoplasm (Figure 3C). Upon Wnt3a induction, wild-type Twa1 and Twa1 mutants containing the CRA domain translocated from the cytoplasm into the nucleus and this restored β-catenin nuclear localization in Twa1-depleted cells (Figures 2F and 3C). However, the Twa1 mutant lacking the CRA domain (Twa1-ΔCRA), which was unable to interact with β-catenin, was mainly localized in the cytoplasm and failed to restore nuclear localization of β-catenin upon Wnt3a stimulation (Figure 3C). Furthermore, Twa1-ΔCRA not only reduced nuclear β-catenin levels (Figure 3D), but also was incapable of enhancing Wnt target gene expression in response to Wnt3a induction (Figure 3E and 3F).

To confirm if Twa1 promotes β-catenin nuclear accumulation through its CRA domain, we forced wild-type or mutant Twa1 to accumulate in the nucleus by fusing a nuclear localization signal (NLS) sequence at their C-terminus. Ectopic expression of nuclear-localized Twa1 (Twa1-NLS) and Twa1 mutants containing the CRA domain not only obviously induced nuclear translocation of exogenous or endogenous β-catenin, but also enhanced Wnt reporter activity and target gene expression even without Wnt3a stimulation (Figure 3G-3J). In contrast, even though the Twa1-CRA-NLS was dominantly localized in the nucleus, it was still unable to increase nuclear β-catenin levels and activate Wnt target gene expression. Collectively, these data suggest that Twa1 facilitates β-catenin nuclear accumulation through its CRA domain.

### Twa1 promotes β-catenin nuclear retention

**Figure 4:**
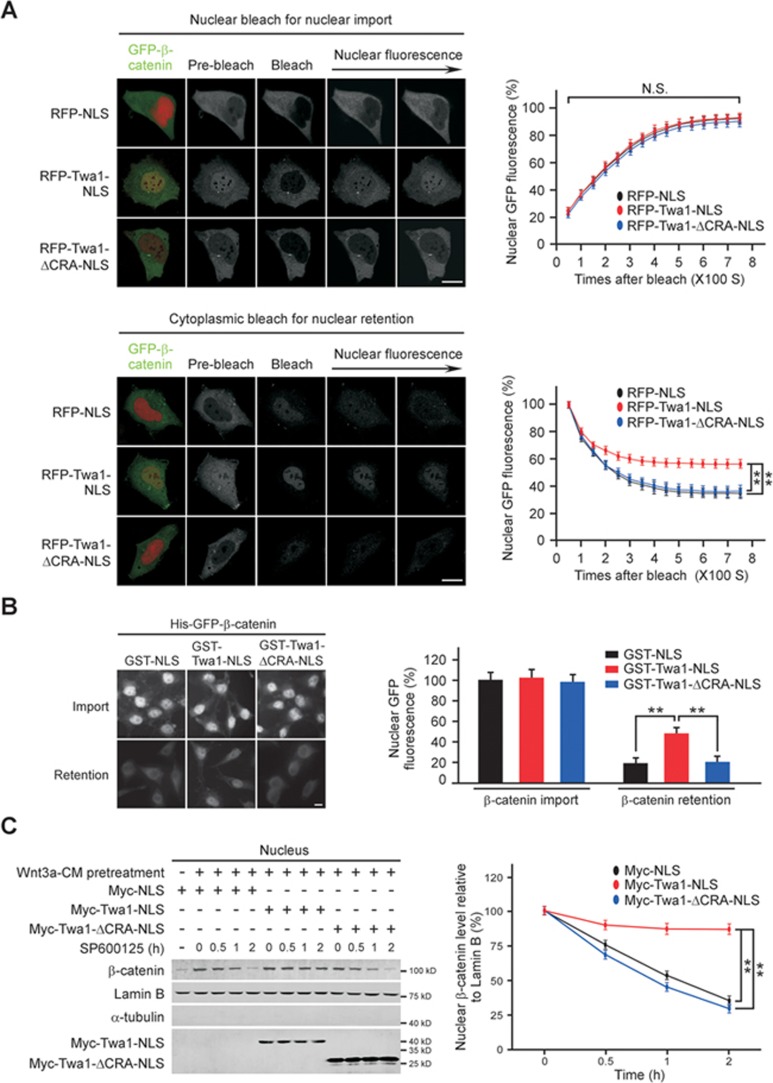
Twa1 promotes β-catenin nuclear retention. **(A)** HEK-293 cells transfected with the indicated constructs were subjected to FRAP analysis. The intensity of nuclear fluorescence was monitored for ∼ 750 s and plotted against time. Scale bars, 10 μm. **(B)** Digitonin-permeabilized cells were subjected to *in vitro* transport assays. For β-catenin import to the nucleus, the permeabilized cells were incubated with the indicated proteins for 20 min and fixed. For β-catenin retention in the nucleus, the cells treated with the indicated proteins for 20 min were washed, incubated with transport buffer for another 20 min, and then fixed. Bars, 10 μm. **(C)** HEK-293 cells transfected with the indicated plasmids were treated with Wnt3a-CM for 4 h, washed to remove Wnt3a-CM, and then treated with SP600125 (10 μM) for the indicated times. Nuclear fractions from the cells were subjected to western analysis. Quantitative data are presented as the mean ± SEM (at least three independent experiments). ns, not significant. ^**^P < 0.01, Student's *t*-test.

To distinguish whether Twa1 facilitates β-catenin nuclear accumulation through either nuclear import or nuclear retention, we performed fluorescence recovery after photobleaching (FRAP) experiments by bleaching GFP-β-catenin in HEK-293 cells expressing RFP-Twa1-NLS (RFP-fused Twa1-NLS) or RFP-Twa1-ΔCRA-NLS (Figure 4A). After photobleaching of GFP-β-catenin in the nucleus, no significant differences were observed in the recovery of nuclear GFP-β-catenin among RFP-NLS-, RFP-Twa1-NLS- and RFP-Twa1-ΔCRA-NLS-transfected cells (Figure 4A), implying that Twa1 does not influence β-catenin nuclear import. In contrast, after photobleaching of cytoplasmic GFP-β-catenin, reduction of nuclear GFP-β-catenin was significantly less in RFP-Twa1-NLS-expressing cells compared with RFP-NLS or RFP-Twa1-ΔCRA-NLS expressing cells, suggesting a role for Twa1 in β-catenin nuclear retention. Further *in vitro* transport assays in digitonin-permeabilized cells revealed that addition of purified Twa1-NLS protein did not affect β-catenin nuclear import but significantly enhanced β-catenin nuclear retention, which was abolished by deletion of the CRA domain (Figure 4B). Moreover, after Wnt3a stimulation and subsequent removal of Wnt3a, ectopic expression of Twa1-NLS, but not Twa1-ΔCRA-NLS, prolonged β-catenin nuclear retention in HEK-293 cells in the presence of SP600125 (a JNK inhibitor to block the nuclear translocation of β-catenin) (Figure 4C)^29^. Together, these results indicate that Twa1 retains β-catenin in the nucleus to promote its nuclear accumulation.

### TCF4 competes with Twa1 for β-catenin binding

**Figure 5:**
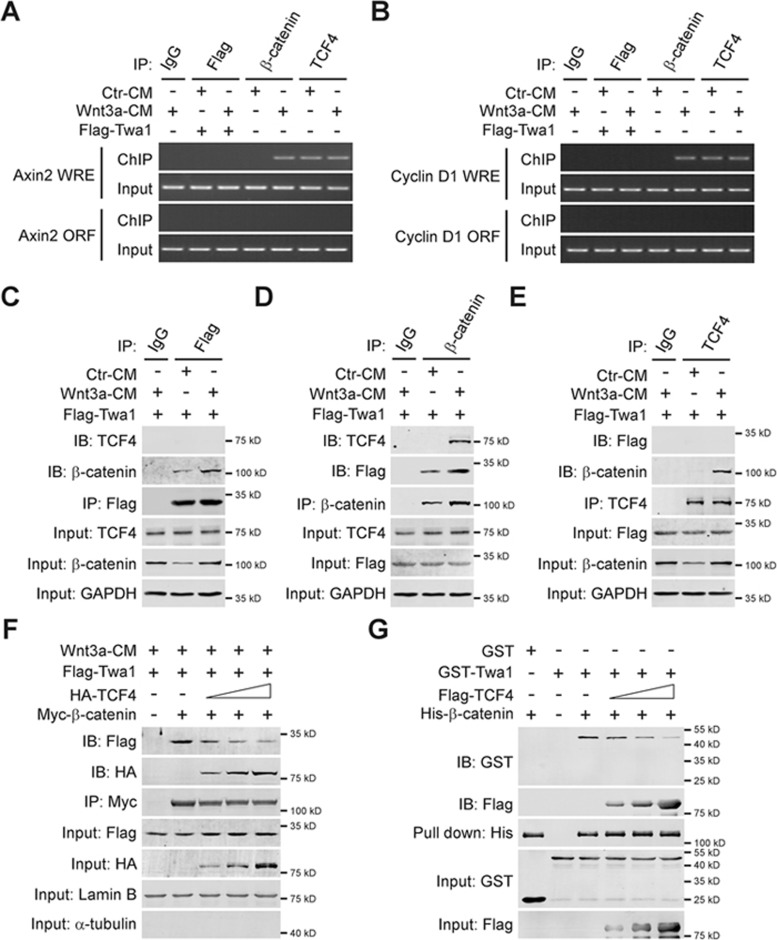
TCF4 competes with Twa1 for β-catenin binding. **(A**, **B)** HEK-293 cells were transfected with the Flag-Twa1 construct or not, treated with Wnt3a-CM or Ctr-CM, and then subjected to ChIP analysis with the indicated antibodies. The ChIP-enriched DNAs were measured by PCR with the primers specific for the *Axin2* or *Cyclin D1* promoter (WREs). The region located in the corresponding ORF (open reading frames) was used as a negative control. **(C-E)** HEK-293 cells transfected with the Flag-Twa1 construct were treated with Wnt3a-CM or Ctr-CM, and then used for co-IP and subsequent western analysis with the indicated antibodies. **(F)** HEK-293 cells transfected with the Flag-Twa1, Myc-β-catenin and increasing amounts of HA-TCF4 plasmids were incubated in Wnt3a-CM for 4 h. The nuclear fractions were extracted and subjected to co-IP analysis using anti-Myc antibody. **(G)** His pull-down analysis of purified His-β-catenin, GST-Twa1 and increasing amounts of Flag-TCF4 protein immunoprecipitated from HEK-293 cells transfected with the Flag-TCF4 construct.

It is well established that nuclear β-catenin associates with TCF4 on WREs to activate target gene expression^4,30^. Given that Twa1 binds and retains β-catenin in the nucleus, we assumed that Twa1 might also directly participate in β-catenin/TCF4-mediated transcription. Chromatin immunoprecipitation (ChIP) analysis showed that only TCF4 was constitutively bound to the WREs of *Axin2* and *Cyclin D1* in the absence of Wnt signals (Figure 5A and 5B). In response to Wnt3a treatment, β-catenin, but not Twa1, was recruited to WREs of *Axin2* and *Cyclin D1*. Further reciprocal co-IP experiments revealed that there was no detectable interaction between Twa1 and TCF4 upon Wnt activation even though β-catenin was associated with both Twa1 and TCF4 (Figure 5C-5E), indicating that Twa1 and TCF4 may form mutually exclusive complexes with β-catenin. To test whether TCF4 competes with Twa1 for β-catenin binding, we performed co-IP experiments in HEK-293 cells and found that the interaction between Twa1 and β-catenin was gradually reduced with increasing expression of TCF4 (Figure 5F). *In vitro* binding experiments further confirmed that when the amount of β-catenin was maintained at constant levels; the more TCF4 associated with β-catenin, the more Twa1 disassociated from β-catenin (Figure 5G). These results strongly suggest that Twa1 does not directly interact with TCF4 and WREs to participate in β-catenin/TCF4-mediated transcription.

### Twa1 is degraded by the Axin complex in the absence of Wnt stimulation

**Figure 6:**
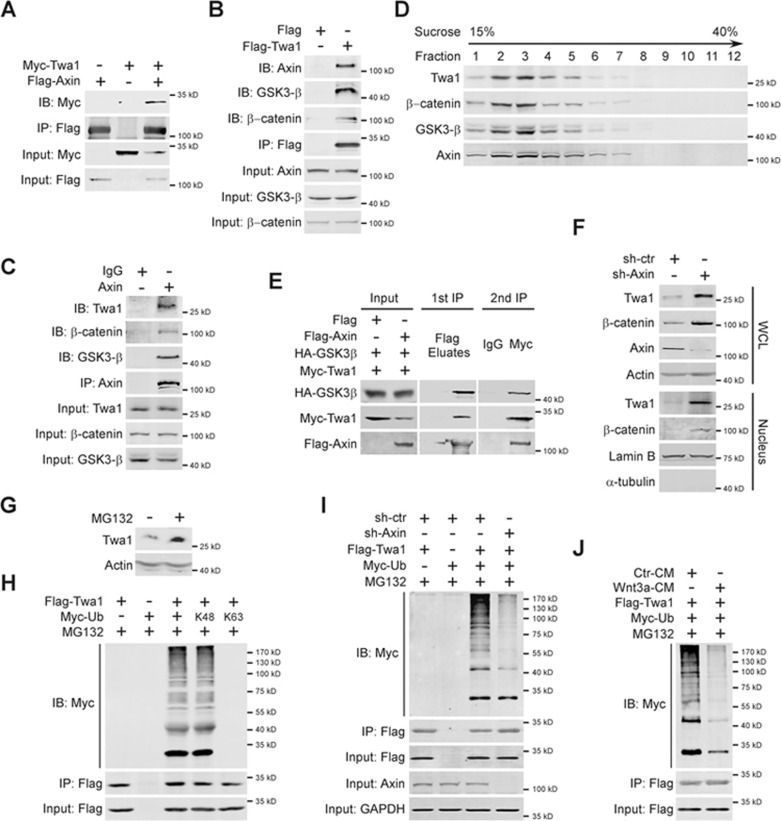
Twa1 is targeted by the Axin complex without Wnt stimulation. **(A**, **B)** HEK-293 cells transfected with the indicated plasmids were processed for co-IP analysis with anti-Flag antibody. **(C**, **D)** Total lysates from HEK-293 cells were subjected to co-IP analysis with anti-Axin antibody **(C)** and sucrose density gradient experiments **(D)**. **(E)** HEK-293 cells transfected with the indicated constructs were used for sequential immunoprecipitation analysis with anti-Flag antibody, followed by anti-Myc antibody. **(F)** Whole-cell lysate (WCL) or nuclear fraction extracted from HEK-293 cells infected with lentiviruses containing sh-Axin or sh-ctr were processed for western blotting with the indicated antibodies. **(G)** HEK-293 cells were treated with MG132 or not, and then used for western analysis. **(H**, **I)** HEK-293 cells infected with lentiviruses containing sh-Axin or sh-ctr were transfected with the indicated plasmids, treated with MG132, and subsequently subjected to co-IP experiments with the indicated antibodies. **(J)** HEK-293 cells transfected with the indicated constructs were treated with MG132, incubated with Wnt3a-CM or not, and then processed for co-IP analysis with the indicated antibodies.

Wnt signaling has been demonstrated to stabilize β-catenin and promote its nuclear translocation through inactivation of the Axin complex^31,32,33^. Since our data showed that Wnt3a treatment increased Twa1 protein level and its nuclear accumulation (Figure 2), we investigated whether Wnt regulates Twa1 via a similar mechanism. Endogenous Twa1 was mainly localized in the cytoplasm of HEK-293 cells without Wnt stimulus. Upon Wnt3a-CM treatment, Twa1 was translocated into the nucleus (Supplementary information, Figure S8). Further co-IP experiments revealed that either exogenous or endogenous Twa1 interacted with components of the Axin complex, including Axin, GSK3 and β-catenin (Figure 6A-6C). In sucrose gradient sedimentation analysis, endogenous Twa1 appeared to co-sediment with Axin, GSK3β and β-catenin (Figure 6D). To test for the simultaneous presence of Axin, GSK3β and Twa1 within the same complex, we performed sequential immunoprecipitation and found that Twa1 formed a complex with Axin and GSK3β (Figure 6E).

To examine whether the Axin complex is involved in the regulation of Twa1 protein levels, we depleted Axin in HEK-293 cells and discovered that knockdown of *Axin* resulted in increased Twa1 levels in whole-cell lysates, as well as in the nuclear fraction (Figure 6F). Knockdown or KO of *APC* also raised Twa1 protein levels and enhanced Twa1 nuclear accumulation (Supplementary information, Figure S9). Since the Axin complex restrains β-catenin levels by promoting its ubiquitination and proteasomal degradation^32,33^, we asked if the Axin complex modulates Twa1 levels in a similar manner. Treatment with the proteasome inhibitor MG132 efficiently increased the protein levels of Twa1 (Figure 6G). In the absence of a Wnt signal, Twa1 was ubiquitinated through lysine 48 (K48)-linked, but not K63-linked, poly-ubiquitin chains (Figure 6H). We next depleted Axin to determine the effect of the Axin complex on Twa1 ubiquitination and discovered that knockdown of *Axin* inhibited Twa1 ubiquitination (Figure 6I), resembling the effect of Wnt3a-CM treatment (Figure 6J). Taken together, these data indicate that in the absence of Wnt signal, Twa1 is targeted by the Axin complex for ubiquitination and degradation.

### Twa1 is essential for zebrafish dorsal development

**Figure 7:**
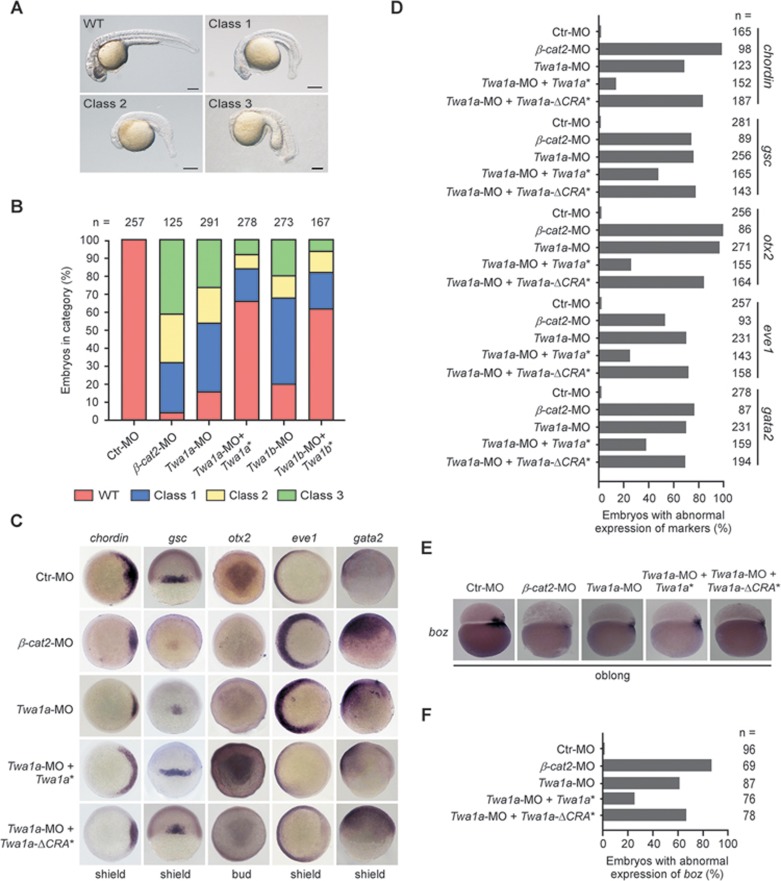
Zebrafish *Twa1* is essential for dorsoventral patterning during embryogenesis. Zebrafish embryos at the one-cell stage injected with the indicated morpholinos targeting *Twa1a* and *Twa1b* (*Twa1a*-MO and *Twa1b*-MO) or mRNAs were harvested at the different times, and subjected to the following analyses. **(A**, **B)** Microscopy showed the different types of embryos at 24 h post-fertilization (hpf). The classes 1-3 represent increasing degrees of phenotypic severity in eye and brain structures. The percentages of embryos with the different classes are shown. **(C-F)** Whole-mount *in situ* hybridization of the dorsal (*gsc*, *chordin* and *otx2*) and ventral (*gata2* and *eve1*) markers and the Wnt target gene *boz*. The percentages of embryos with abnormal expression of the indicated markers are also presented. *β-Cat2*, *β-catenin 2*; *boz*, *bozozok*; n, number of observed embryos; Twa1a^*^, zebrafish *Twa1a*-MO-resistant mRNA; *Twa1a*-ΔCRA^*^, zebrafish *Twa1a*-MO-resistant mRNA without CRA domain; Twa1b^*^, zebrafish *Twa1b*-MO-resistant mRNA.

To assess whether Twa1 plays a role in Wnt signaling *in vivo*, we extended our analyses to zebrafish embryos, where nuclear β-catenin is required for dorsal development^34,35^. In zebrafish, *Twa1* has two homologous genes: *Twa1a* and *Twa1b* (Supplementary information, Figure S10A). Both *Twa1a* and *Twa1b* were ubiquitously expressed in the early developmental stages of zebrafish embryos (Supplementary information, Figure S10B and S10C). We therefore injected zebrafish embryos at the one-cell stage with *Twa1a*-morpholino (*Twa1a*-MO) or *Twa1b*-MO to knock down the expression of these two proteins. We found that both *Twa1a* and *Twa1b* morphants resembled *β-catenin 2* morphants and exhibited loss or reduction of dorsal structures, characteristics of embryonic ventralization (Figure 7A and 7B; Supplementary information, Figure S11)^36,37^. Moreover, the *Twa1a* morphants showed reduced expression of the dorsal markers *chordin*, *goosecoid* and *otx2*, and expanded expression of the ventral markers *eve1* and *gata2* (Figure 7C and 7D)^38^. To test if these defects were caused by Wnt signaling deficiency, we examined the expression of the maternal Wnt target gene *bozozok* (*boz*)^39,40^, and found that depletion of *Twa1a* markedly decreased *boz* expression (Figure 7E and 7F). Importantly, all of these ventralized phenotypes were efficiently reversed by morpholino-resistant *Twa1* mRNA, but not by morpholino-resistant *Twa1*-Δ*CRA* mRNA (Figure 7). Thus, these results imply that Twa1 is essential for Wnt signaling during zebrafish dorsal development.

### Twa1 promotes the proliferation of CRC cells

**Figure 8:**
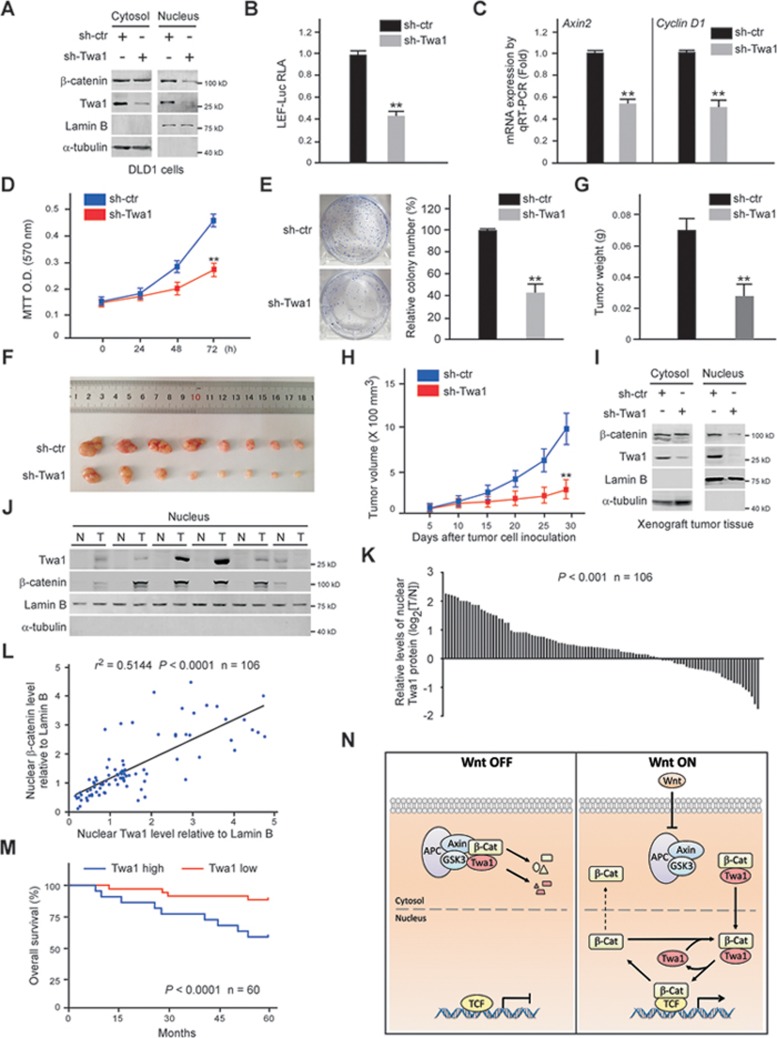
Nuclear Twa1 is associated with CRC cell proliferation and poor prognosis of CRC patients. **(A-E)** DLD1 cells infected with lentiviruses containing sh-Twa1 or sh-ctr were subjected to immunoblotting **(A)**, dual luciferase reporter activity **(B)**, Wnt target gene expression **(C)**, MTT **(D)** and colony formation analyses **(E)**. Lamin B and α-tubulin were used as loading controls for nuclear and cytosolic fractions, respectively. Quantitative data are presented as the mean ± SEM (at least three independent experiments). ^**^P < 0.01, Student's *t*-test. **(F-I)** DLD1 cells infected with lentiviruses containing sh-Twa1 or sh-ctr were subcutaneously injected into nude mice. Representative tumors dissected at 28 days post injection are shown **(F)**. Graphs indicate tumor weights **(G)** and the growth curve of tumor volumes **(H)**. The cytosolic and nuclear fractions from xenograft tumors were subjected to western analysis with the indicated antibodies **(I)**. Quantitative data are expressed as the mean ± SEM. ^**^P < 0.01, Student's *t*-test. **(J-L)** The expression of nuclear Twa1 and β-catenin in human CRC tissues compared to their corresponding nontumor tissues. Representative images of western blotting show nuclear levels of Twa1 and β-catenin **(J)**. The densities of bands were quantified by Image J software and normalized to lamin B. Data are presented as log_2_ value of Twa1 (T/N). *P* < 0.001, Student's *t*-test **(K)**. The correlation between nuclear Twa1 and β-catenin levels was determined by linear regression test (*P* < 0.0001) **(L)**. **(M)** Kaplan-Meier survival curves for patients with high and low levels of nuclear Twa1 in human CRC tissues. *P* < 0.0001, log rank test. **(N)** Working model for the regulation of β-catenin nuclear retention by Twa1 in canonical Wnt signaling. β-Cat, β-catenin.

Since aberrantly elevated nuclear β-catenin is a hallmark of oncogenic Wnt signaling in colorectal tumorigenesis^5,41^, we examined the role of Twa1 in a model of this pathological process. Silencing Twa1 in CRC cells (DLD1 and SW480 cells), which harbor constitutively high levels of nuclear β-catenin due to *APC* mutations^42,43^, significantly reduced nuclear β-catenin levels, Wnt reporter activity, and target gene expression (Figure 8A-8C; Supplementary information, Figure S12A-S12C). Importantly, Twa1 depletion also significantly suppressed β-catenin nuclear retention, but did not influence nuclear import, in CRC cells (Supplementary information, Figure S13). Given that oncogenic Wnt activation is essential for CRC cell proliferation^5,44^, we performed MTT assays and colony formation experiments, and observed that depletion of Twa1 significantly inhibited cell proliferation and colony formation in CRC cells (Figure 8D and 8E; Supplementary information, Figure S12D and S12E). To further study the function of Twa1 in tumor growth *in* *vivo*, we used a xenograft model with CRC cells, and found that *Twa1* knockdown significantly suppressed xenograft tumor growth with concomitant decreases in nuclear β-catenin levels (Figure 8F-8I). Collectively, these findings suggest that Twa1 plays an indispensable role in oncogenic Wnt signaling and proliferation of CRC cells.

### Nuclear Twa1 is significantly associated with CRC prognosis

To explore the clinical significance of nuclear Twa1 in CRC, we examined the levels of nuclear Twa1 in CRC tissues. Nuclear extracts were prepared from 106 pairs of CRC samples and their matched nontumor tissues. Western analysis revealed that nuclear Twa1 was significantly upregulated in CRC tissue samples compared with control tissues (*P* < 0.001) (Figure 8J and 8K; Supplementary information, Figure S14). Furthermore, the levels of nuclear Twa1 highly correlated with those of nuclear β-catenin in CRC tissues (*r*^2^ = 0.5144, *P* < 0.0001) (Figure 8L). More importantly, patients with higher levels of nuclear Twa1 in their tumors showed a shorter overall survival time (*P* < 0.0001) (Figure 8M). Taken together, these results indicate that nuclear Twa1 is associated with poor prognosis of CRC patients.

## Discussion

In this study, we provide evidence that Twa1 is a critical β-catenin nuclear retention factor during Wnt signaling. In the absence of Wnt signals, both Twa1 and β-catenin are degraded via ubiquitination by the Axin complex. In response to Wnt stimulation, both cytoplasmic Twa1 and β-catenin are stabilized and translocated into the nucleus, where Twa1 retains β-catenin to ensure a sufficient amount of β-catenin for TCF4 binding, but does not directly participate in β-catenin/TCF4-mediated transcription (Figure 8N). We exemplify the roles of Twa1 in development by showing its requirement for dorsal development of zebrafish embryos. This further supports a role for Twa1 in mediating the nuclear retention of β-catenin as an essential part of Wnt signaling *in vivo*. Thus, Twa1 is a previously uncharacterized component of the Wnt signaling pathway that promotes β-catenin nuclear retention.

The molecular regulation of β-catenin nuclear accumulation during Wnt signaling remains enigmatic. Several steps have been implicated to be necessary for the nuclear accumulation of β-catenin: stabilization of β-catenin in the cytoplasm, its transport into the nucleus and subsequently its retention in the nucleus^1,2^. β-catenin does not contain NLS or nuclear export signal (NES) sequences^1,2^ and earlier studies have shown that β-catenin enters into the nucleus by directly interacting with the nuclear pore complex, independently of the Ran GTPase or importin-mediated mechanism^45,46,47,48^. Recent reports demonstrate that some NLS-containing proteins, including mucin-1, IRS-1, BCL9 and FoxM1, are able to directly bind to β-catenin and transport β-catenin into the nucleus via the classical import pathway^7,8,9,10^. On the other hand, some NES-containing proteins, such as APC, Axin and Chibby, have been shown to associate with β-catenin and promote the export of nuclear β-catenin^11,12,13,14,15,16^.

It is well-established that nuclear β-catenin directly binds to TCF family members such as TCF4 and LEF1 to activate Wnt target gene expression^1,2^. Overexpression of TCF4 shifts ectopic cytoplasmic β-catenin to the nucleus and slows its rate of nucleocytoplasmic shuttling^49,50^. Downregulation of LEF1 selectively reduces the chromatin-retained pool of nuclear β-catenin in NIH-3T3 cells^50,51^. Although nuclear β-catenin is captured by the chromatin-bound TCF family members on chromatin, recent kinetic ChIP analyses have shown that unlike TCF4 which maintains a steady state on WREs, β-catenin dynamically cycles on and off WREs during Wnt activation^52,53^. It is unclear how nuclear β-catenin that is dissociated from chromatin is retained in the nucleus to maintain sufficient levels for TCF4 binding.

Here our data clearly show that Twa1 suppresses β-catenin nuclear export, but has no significant effect on its nuclear import (Figure 4). Although Twa1 interacts with β-catenin in the nucleus, we did not detect the interaction between Twa1 and either TCF4 or WREs (Figure 5A-5E), suggesting that Twa1 and TCF4 may form mutually exclusive complexes with β-catenin. Moreover, if the amount of β-catenin is kept constant, the more TCF4 associates with β-catenin, the more Twa1 disassociates from β-catenin (Figure 5F and 5G). Taken together, these results indicate that Twa1 does not directly participate in β-catenin/TCF4-mediated transcription, but only retains β-catenin in the nucleus. This mechanism may ensure that sufficient nuclear β-catenin is available for TCF4 binding through previously unrecognized chromatin-independent retention for Wnt signaling. It is possible that Twa1 could function as a scaffold protein to bridge β-catenin to other nuclear retention factors, or that it could inhibit the nuclear export of β-catenin by interfering with the action of nuclear export factors. Future work on identifying the factors that function together with Twa1 will provide insight into how Twa1 promotes the nuclear retention of β-catenin.

Previous studies have reported Twa1 to be a partner of RanBPM in a yeast two-hybrid assay^17,18^. RanBPM functions as a scaffolding protein in several signaling pathways and mediates multiple cellular processes^54,55^. However, unlike most members of the Ran-binding protein family, RanBPM does not contain the consensus domain for Ran binding *in vivo*^56^, consistent with its lack of involvement in nuclear-cytoplasmic shuttling^55,57^. Nevertheless, it will still be of interest to examine whether RanBPM participates in Twa1-mediated nuclear retention of β-catenin.

In human CRC, aberrant Wnt activation is usually caused by mutations in genes encoding Wnt signaling components that increase the stability and nuclear levels of β-catenin^58^. A high level of nuclear β-catenin is frequently found in CRC tissues and is correlated with poor prognosis of CRC patients^59,60^. Our results indicate that Twa1 is an essential β-catenin nuclear retention factor that contributes to colorectal carcinogenesis. Twa1 deficiency not only decreases β-catenin nuclear levels and Wnt target gene expression in CRC cells, but also inhibits the proliferation and tumorigenicity of CRC cells in nude mice (Figure 8A-8I; Supplementary information, Figure S12). More importantly, nuclear Twa1 is highly expressed in human CRC tissues and this significantly correlates with nuclear β-catenin levels and the poor survival of CRC patients (Figure 8J-8M), suggesting that Twa1 may be a potential new target for CRC therapy.

## Materials and Methods

### Bioinformatics analysis

To identify differentially expressed genes in CRC, we analyzed the Hong CRC microarray data set (GSE9348, http://www.oncomine.org) available in the Oncomine database. The cohort of human CRC tissues profiled on Affymetrix U133 Plus 2.0 arrays were normalized with the Robust Multi-array Average algorithm. The limma R package (v 3.2.3) was used to detect differentially expressed genes. The Linear models and Empirical Bayes methods were applied for analyzing these data, and the resulting *P*-values were adjusted using the Benjamini and Hochberg FDR algorithm^61^. Differentially expressed genes were thresholded at fold change > 2, *P* < 0.01 and FDR < 0.01.

To confirm Twa1's upregulation in CRC tissues, we analyzed the CRC RNA-seq data set (level 3 data of all pair-matched CRC tissues from Illumina GA and HiSeq platforms) obtained from the TCGA databases (http://cancergenome.nih.gov). RNA-seq by Expectation-Maximization (RSEM) expression values were log2 transformed and used for statistical analysis with Student's *t*-test^62^.

### Constructs

Full-length human *Axin*, *β-catenin*, *TCF4*, *Twa1*, zebrafish *Twa1a* and *Twa1b* were amplified from total RNA by RT-PCR and cloned into the pEF-Flag vector or the pCS2+ vector with or without an N-terminal Flag, HA, or Myc tag. The *Twa1-ΔLisH* mutant lacking the amino acids (aa) 27-59, the *Twa1-ΔCTLH* mutant deleted for amino-acids 65-122 and the *Twa1-ΔCRA* mutant deleted for amio-acids 118-215 were generated by PCR and subcloned into the pCS2+ vector containing an N-terminal Flag tag or the pCMV/myc/nuc vector containing a C-terminal NLS (Invitrogen). Myc-Ub, Myc-Ub-K48, Myc-Ub-K63, pCMV-Flag-YAP, pCMV-Flag-TAZ, pEF-Flag, pEP330X, pCS2+, LEF-Luc reporter, GLI-Luc reporter, NF-κB-Luc reporter, TEAD-Luc reporter, GFP-β-catenin and HA-GSK3β vectors were provided by Drs Yong Cang, Zongping Xia, Jinrong Peng, Ximei Wu (Zhejiang University), W James Nelson (Stanford University) or Jim R Woodgett (Toronto University). The luciferase reporters of various signaling cascades were constructed based on pGL3-basic vectors (Promega) as described previously^33,63^. Oligos corresponding to the following sequences were synthesized and cloned into pGV3-U6 (a lentivirus-based RNAi vector, Genepharma), separately: 5′-GGAGAAGTTTCGAATGGAA-3′ and 5′-CAGCGGAGAAGTTTCGAAT-3′ for Twa1 RNAi (sh-Twa1 and sh-Twa1-2), 5′-GTACATTCTTGATAACAAT-3′ for Axin RNAi (sh-Axin), 5′-GGTGGTGGTTAATAAGGCT-3′ for β-catenin RNAi (sh-β-catenin) and 5′-GACGTTGCGAGAAGTTGGA-3′ for APC RNAi (sh-APC). Twa1 RNAi-resistant mutants were generated with a QuickChange Site-Directed Mutagenesis kit (Stratagene). All of these constructs were confirmed by DNA sequencing.

### Cell culture, transfection and lentiviral infection

HEK-293, HEK-293T, HeLa, Wnt3a-expressing and control L cells were cultured in DMEM (Invitrogen) containing 10% fetal bovine serum (FBS, Gibco). DLD1 and SW480 cells were grown in RPMI1640 (Invitrogen) containing 10% FBS. Wnt3a-conditioned medium (Wnt3a-CM) and control medium (Ctr-CM) were prepared as previously described^29^. Shh-conditioned medium (Shh-CM) was provided by Dr Ximei Wu (Zhejiang University). Wnt3a-expressing and L cells were purchased from American Type Culture Collection. The other cells were obtained from the Cell Bank of the Chinese Academy of Science (Shanghai, China).

Transient transfection in HEK-293, DLD1 and SW480 cells were carried out using the Lipofectamine 2000 reagent (Invitrogen) according to the manufacturer's instructions. Lentiviruses were prepared in HEK-293T cells by transfection with the indicated shRNA vectors and viral packaging constructs. The viral medium was collected, filtered and mixed with 4 μg/ml polybrene (Sigma) before addition to the target cells.

### Generation of *Twa1* or *APC* knockout cells by CRISPR/Cas9

The *Twa1* gene was inactivated in HEK-293 cells using the CRISPR/Cas9 system. Briefly, a pair of targeting sequences (5′-GAGAGCAGACATGAACCGCC-3′) was synthesized and introduced into a modified one-vector system pEP330X. HEK-293 cells transfected with this plasmid were treated with 1 μg/ml puromycin for 24 h and reseeded to 96-well plates to allow single-colony formation. After 12 days, genomic DNA was extracted from individual colonies. The candidate KO clones were verified by sequencing of the PCR fragments amplified by the primers (5′-ATTCTCCGGCTCACAGCTC-3′ and 5′-GCTACAGCACTCCTTATGTGTT-3′) and western blotting with anti-Twa1 antibody. The *APC* KO HEK-293 cells were prepared as previously described^64^. The candidate KO clones were verified by sequencing of the PCR fragments amplified by the primers (5′-AAACTCATTTGGCCCACAGG-3′ and 5′-TGCTTTGAAACATGCACTACGA-3′).

### Luciferase reporter assay

HEK-293 cells infected with lentiviruses containing sh-Twa1 or sh-ctr were seeded in 12-well plates at 2.5 × 10^4^ cells per well and then transfected with LEF-Luc (200 ng) and Tk-renilla (10 ng, Promega) vectors for 24 h. After treatment with Wnt3a-CM or 40 mM LiCl (Sigma) for 6 h, the cells were washed twice with phosphate buffered saline (PBS) and lysed in 200 μl passive lysis buffer (Promega). The luciferase activities were measured using dual luciferase reporter system (Promega). Firefly luciferase activities were normalized to Renilla luciferase activities.

### Immunoflourescence microscopy

Immunoflourescence analysis was performed as described previously^65^. Cells grown on coverslips were fixed with 4% paraformaldehyde for 15 min, and blocked with 10% FBS for 30 min. The coverslips were then incubated with the indicated antibodies for 2 h, followed by Cy3-conjugated anti-mouse Ig or FITC-conjugated anti-rabbit Ig secondary antibody (Jackson Immuno Research) for 50 min. DNA was stained with 4′, 6-diamidino-2-phenylindole (DAPI, Sigma). The mounted coverslips were analyzed by Zeiss LSM510 microscopy.

### FRAP analysis

FRAP assays were performed in live cell chambers (Corning) at 37 °C using a Zeiss LSM510 microscopy. HEK-293, SW480 or DLD1 cells expressing GFP-catenin were bleached by the 488 nm laser line of the 20 mW argon laser at 100% power. About 90% of either nuclear or cytoplasmic GFP signal was bleached. Images were then taken with 35 frames at 25-s intervals. Average intensities in regions of interest were measured using MetaMorph software (Molecular Devices). The intensity in the pre-bleach image was set to 100%, and the first post-bleach image was set as time point 0. The recovery curves shown are averages of at least six cells (at least three independent experiments).

### *In vitro* transport assay

Digitonin-permeabilized HeLa cells were prepared as described previously^66^. The transport assay was performed in testing solution (1 mM GTP, 1 μM His-Ran, 0.2 μM His-Importin-α, 0.2 μM His-Importin-β, 1 μM His-GFP-β-catenin) containing an energy-regenerating system (1 mM ATP, 5 mM phosphocreatine, 20 units of creatine kinase) and transport buffer (20 mM HEPES, pH 7.3, 2 mM magnesium acetate, 110 mM potassium acetate, 5 mM sodium acetate, 2 mM dithiothreitol, 0.5 mM EGTA, 1 g/ml aprotinin, leupeptin and pepstatin A). For β-catenin import to the nucleus, the cells were incubated in the testing solution with the indicated proteins (1 μM GST-NLS, GST-Twa1-NLS or GST-Twa1-ΔCRA-NLS) for 20 min and then fixed with 4% formaldehyde. For β-catenin retention in the nucleus, cells were treated with testing solution and the indicated proteins for 20 min, washed, rinsed with the transport buffer for another 20 min, and then fixed. The GFP intensity of each nucleus was measured by fluorescence microscopy (Olympus IX71). The mean intensity of 40 nuclei was plotted. The intensity of GFP signal in the nuclei incubated with His-GFP-β-catenin and GST-NLS in the import assay was set as 100%.

### MTT and colony formation assays

For MTT (3-(4,5-dimethyl-2-thiazolyl)-2,5-diphenyl-2-H-tetrazolium bromide) assays, DLD1 or SW480 cells (3 × 10^3^ per well) were seeded in 96-well culture plates for 24 h and incubated with MTT (5 mg/ml, 20 μl) for 2 h. Dimethyl sulfoxide (100 μl) was then added into each well and resuspended until all crystals had been dissolved. The absorbance of the samples was measured with a spectrophotometer at 570 nm^67^.

For colony formation assays, DLD1 or SW480 cells were seeded at a density of 1 × 10^3^ cells per 35 mm diameter dishes. After 14 days, the cells were stained with 0.5% crystal violet in 20% ethanol for 10 min. The number of colonies was photographed and determined by Image J software (NIH).

### Quantitative RT-PCR analysis

Single-stranded cDNA was synthesized from total RNA using AMV reverse transcriptase (Takara). qRT-PCR was performed using SYBR Green PCR Master Mix (Takara) and 7500 Real-Time PCR System (Applied Biosystems). The primers for qRT-PCR of *Twa1*, *Axin2*, *Cyclin D1*, *GAPDH* and *U6* are listed: *Twa1*, 5′-CTGGAAACACTTGATGAACG-3′ and 5′-ATCTCTGTGAGGCACTCTCG-3′ *Axin2*, 5′-CTGGCTCCAGAAGATCACAAAG-3′ and 5′-ATCTCCTCAAACACCGCTCCA-3′ *Cyclin D1*, 5′-AGCTCCTGTGCTGCGAAGTGGAA-3′ and 5′-AGTGTTCAATGAAATCGTGCGGG-3′ *APC*, 5′-GTCCAAGGGTAGCCAAGGATG-3′ and 5′-CATCCTTGGCTACCCTTGGAC-3′ *GAPDH*, 5′-GCACCACCAACTGCTTA-3′ and 5′-AGTAGAGGCAGGGATGAT-3′ *U6*, 5′-CTCGCTTCGGCAGCACA-3′ and 5′-AACGCTTCACGAATTTGCGT-3′.

### Antibodies

Anti-β-catenin (BD Bioscience), Twa1, lamin B (Proteintech), GSK3β, TCF4, Axin1 (Cell Signal Technology), Myc, GAPDH, GST, His, HA, Flag (Santa Cruz), actin and α-tubulin (Sigma) antibodies were acquired commercially.

### Western blotting

Lysates from cells or tissues were prepared in a radioimmunoprecipitation assay (RIPA) buffer (50 mM Tris, pH 7.5, 1% Triton X-100, 0.5% deoxycholate, 10 mM EDTA, 150 mM NaCl) containing 50 mM NaF, 1 mM sodium vanadate, 1 μg/ml leupeptin, 0.1 μg/ml aprotinin and a cocktail of protease inhibitors (Roche), and then subjected to western analyses with the indicated antibodies and IRDye 700CW- or 800CW-conjugated secondary antibodies. The IRDye 700CW or 800CW activities were detected by the LI-COR Odyssey system (LI-COR Biosciences). Cytosolic and nuclear fractions were prepared using a Nuclear Extract Kit (Active Motif) according to the manufacturer's instructions. GAPDH, lamin B, α-tubulin and actin were used as loading controls for western analysis.

### Pull-down assays

Pull-down assays were performed as described^68,69^. In brief, GST, GST-Twa1, GST-Twa1-ΔLisH, GST-Twa1-ΔCTLH, GST-Twa1-ΔCRA or His-β-catenin expressed in *Escherichia coli* BL21 were purified and incubated in Tris buffered saline Nonidet P-40 buffer (20 mM Tris, pH 8.0, 150 mM NaCl, 0.5% NP-40, 5 mM EGTA, 1.5 mM EDTA, 0.5 mM Na_3_VO_4_, 20 mM p-nitrophenyl phosphate) supplemented with a cocktail of protease inhibitors at 4 °C for 4 h, and then added by glutathione-agarose beads (GST pull-down) or Ni-NTA-agarose beads (His pull-down) for another 2 h. The bound proteins were resolved by SDS-PAGE and subjected to western blotting.

### Co-IP experiments

Cell lysates were incubated with the indicated antibodies at 4 °C overnight and added with 50 μl Protein A/G Sepharose beads (Santa Cruz) for another 4 h. The immunoprecipitates were washed and then processed for western blotting.

For the sequential IP assay, lysates from HEK-293 cells transfected with HA-GSK3β, Myc-Twa1 and Flag-Axin were incubated with anti-Flag antibody bound to protein A/G-agarose beads at 4 °C overnight. The immunoprecipitates were washed and then eluted with Flag peptide for 2 h at 4 °C. The Flag eluates were subsequently incubated with anti-Myc antibody or control IgG at 4 °C overnight and added with protein A/G-agarose beads for another 4 h. The immunoprecipitates were washed and then processed for western blotting.

For the ubiquitination assays, the cells were treated with 25 μM MG132 (Sigma) for 2 h before collection and lysed by RIPA buffer with 10 mM N-ethylmaleimide (Sigma) and a cocktail of protease inhibitors. The prepared lysates were then immunoprecipitated with anti-Flag antibody at 4 °C overnight and subjected to western analysis.

### Sucrose gradient sedimentation

Sucrose gradient sedimentation was carried out as previously described^70^. Briefly, HEK-293 cells were harvested in Hank's buffer on ice, pelleted and lysed for 20 min in extraction buffer (30 mM Tris, pH 7.3, 140 mM sodium chloride, 25 mM sodium fluoride, 1% Triton X-100) containing 3 mM sodium ortho-vanadate, 2 mM phenylmethylsulfonyl fluoride, 100 nM okadaic acid and a cocktail of protease inhibitors. The supernatant was layered on top of a 15%-40% sucrose gradient with a buffer containing 30 mM Tris (pH 7.3), 0.02% Triton X-100, 140 mM sodium chloride, 25 mM sodium fluoride, 3 mM sodium ortho-vanadate and a cocktail of protease inhibitors. Ultracentrifugation was performed in a Beckman SW55 rotor at 100 000× *g* for 4 h at 4 °C. After centrifugation, fractions were collected from the bottom of the tube and subjected to western analysis.

### ChIP analysis

For ChIP assays, 3 × 10^6^ cells were prepared with the ChIP assay kit (Cell Signaling Technology) according to the manufacturer's instructions. The resulting precipitated DNA was analyzed by PCR as described previously^7,71^.

### Zebrafish embryo manipulation and *in situ* hybridization

Wild-type zebrafish (strain AB) were maintained at 28.5 °C using standard protocols. Procedures for animal staging and injection were performed as described^37,38,39,40^. Synthetic RNAs were transcribed *in vitro* using pCS2-Twa1a, Twa1b or Twa1a-ΔCRA vectors with MessageMachine (Ambion). All morpholinos (MOs) were purchased from Gene Tools. The following targeting sequences were used: *Twa1a*: 5′-CTGGCTTTTCAGCATAACTCATCAT-3′ *Twa1b*: 5′-CAGGCTTTTCTGAATAGCTCATCACA-3′ *β-catenin 2*: 5′-AGCCATCGTTGCGTCAATCCTTTAG-3′ standard control: 5′-CCTCTTACCTCAGTTACAATTTATA-3′. The doses of each MO and mRNA for injection were 6 ng and 15 pg, respectively.

For *in situ* hybridization, the sequences of probes (*boz*, *chordin*, *eve1*, *gata2*, *gsc* and *otx2*) were amplified from zebrafish cDNAs and cloned into pCS2+ vectors^37,38,39,40^. All constructs were confirmed by DNA sequencing. The probes were labeled using the Digoxigenin labeling kit (Roche). Whole-mount *in situ* hybridization was carried out as described previously^37,38,39,40^.

### Xenograft tumor formation

All mouse experiments were conducted in accordance with the Guide for the Care and Use of Animals for research purposes, and were approved by the Committee of Animal Ethics, Zhejiang University. Four-week-old female nude mice were bred in a specific pathogen-free environment in the Animal Facility, Zhejiang University. DLD1 cells were infected by lentiviral particles for 3 days, and then treated with 2 μg/ml puromycin for 1 day. The puromycin-resistant cells were trypsinized, washed and resuspended in PBS/Matrigel (1:1) and subcutaneously injected into the left flank of nude mice (4 × 10^6^ cells per mouse). Tumor diameters were serially measured with calipers every 5 days, and tumor volumes were calculated using the formula *V* = (*L×W^2^*)/2, where *V* = volume (in mm^3^), *L*= length (in mm), *W* = width (in mm). The mice were killed and the tumors were isolated at 28 days after inoculation for further analysis.

### CRC samples

CRC tumor tissues and their pair-matched nontumor tissues from 106 patients undergoing resection for CRC were obtained from the Zhejiang Cancer Hospital and the Second Affiliated Hospital, Zhejiang University School of Medicine (Hangzhou, China). All participants provided written informed consent for this study and the Ethics Committee of Zhejiang University School of Medicine approved the protocol. The clinical data were also obtained from medical records of the above hospitals. The clinicopathological characteristics of included patients were independently evaluated by at least two professional pathologists and summarized in Supplementary information, Figure S14.

### Statistical analysis

All experiments were repeated at least three times and representative images are shown. The two-tailed Student's *t*-tests were used for comparisons between two groups. A linear regression test was used to determine significance of the correlation between nuclear Twa1 and β-catenin levels. A log-rank test was used to determine significance for Kaplan-Meier analysis. *χ*^2^-tests were performed to determine significance of the relationship between expression of nuclear Twa1 and clinicopathologic features in CRC patients. A *P*-value < 0.05 was considered statistically significant.

## Author Contributions

TZ, YL and SX designed the experiments. YL, SX, WZ, CZ, CG, QS, YC and ZX performed the experiments. TZ and YL supervised direction of project, conducted experiments, interpretation of data and wrote the manuscript. All authors especially YK discussed the results and commented on the manuscript.

## Competing Financial Interests

The authors declare no competing financial interests.

## Supplementary Information

Supplementary information, Figure S1 Bioinformatics analysis of gene expression in 70 human CRC tissues (T) and 12 nontumor tissues (N) from the Hong CRC microarray dataset (GSE9348) available in the Oncomine database.

Supplementary information, Figure S2 Twa1 is selectively involved in canonical Wnt signaling.

Supplementary information, Figure S3 Knockdown of *Twa1* reduces the protein levels of endogenous Twa1 in HEK-293 cells.

Supplementary information, Figure S4 Twa1 facilitates β-catenin nuclear accumulation and Wnt target gene expression in RKO cells.

Supplementary information, Figure S5 Twa1 promotes β-catenin nuclear accumulation and Wnt target gene expression in response to LiCl treatment.

Supplementary information, Figure S6 Knockout of *Twa1* suppresses β-catenin nuclear accumulation and Wnt activation.

Supplementary information, Figure S7 Twa1 binds to β-catenin *in vitro* and *in vivo*.

Supplementary information, Figure S8 Subcellular localization of endogenous Twa1 in HEK-293 cells.

Supplementary information, Figure S9 Either knockout or knockdown of *APC* gene increases Twa1 protein levels and its nuclear accumulation in HEK-293 cells.

Supplementary information, Figure S10 Characterization of zebrafish homologues of *Twa1*, *Twa1a* and *Twa1b*.

Supplementary information, Figure S11 Effectiveness of the morpholinos targeting *Twa1a* and *Twa1b* mRNA (*Twa1a*-MO and *Twa1b*-MO) in zebrafish embryos.

Supplementary information, Figure S12 Depletion of Twa1 inhibits Wnt activation and cell proliferation in SW480 cells.

Supplementary information, Figure S13 Twa1 is required for β-catenin nuclear retention in colon cancer cells.

Supplementary information, Figure S14 Relationship between nuclear Twa1 levels and clinicopathologic features in CRC patients.
