# Supplementary material for: Twa1/Gid8 is a β-catenin nuclear retention factor in Wnt signaling and colorectal tumorigenesis
Source: Cell Res. 2017 Aug 22;27(12):1422–40. doi: 10.1038/cr.2017.107 (PMC5717399; doi:10.1038/cr.2017.107)
Supplement: Supplementary information, Figure S1 — Bioinformatics analysis of gene expression in 70 human CRC tissues (T) and 12 nontumor tissues (N) from the Hong CRC microarray dataset (GSE9348) available in the Oncomine database. [file cr2017107x1.pdf]

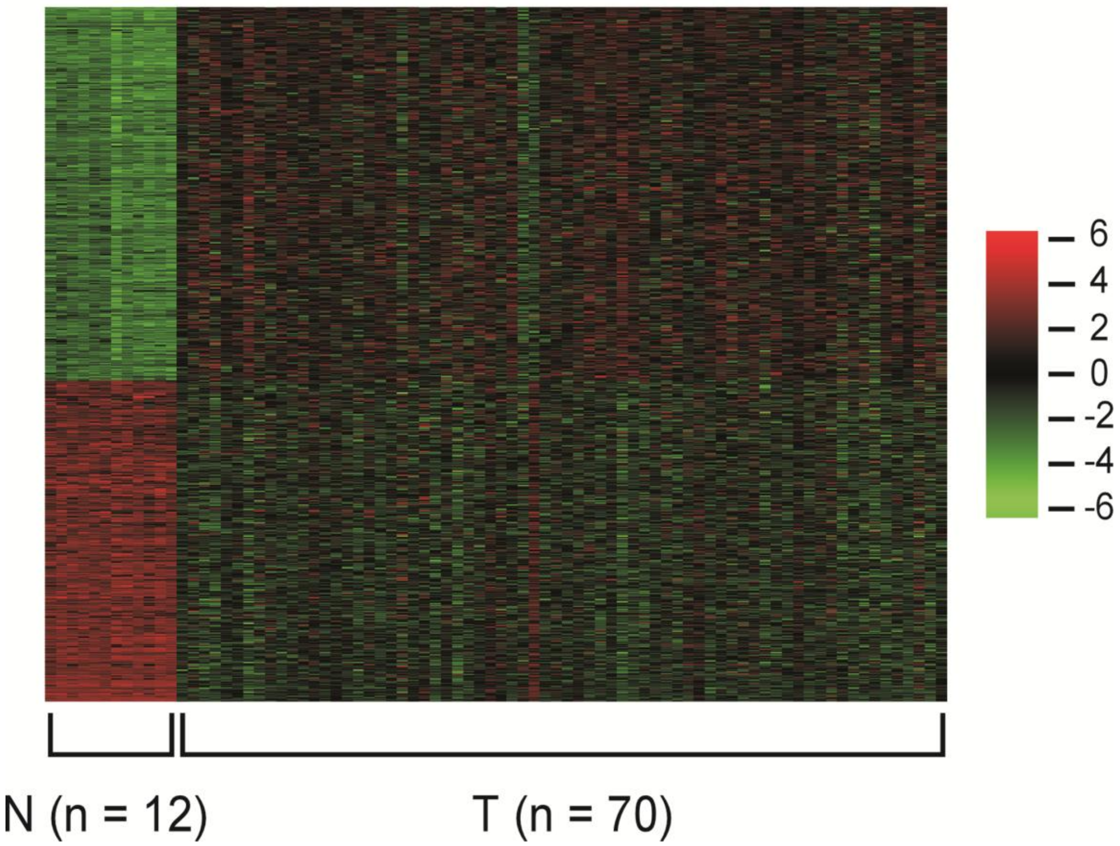
6
4
2
0
-2
-4
-6
N (n = 12)
T (n = 70)

Supplementary information, Figure S1 Bioinformatics analysis of gene expression in 70 human CRC tissues (T) and 12 nontumor tissues (N) from the Hong CRC microarray dataset (GSE9348) available in the Oncomine database. The heatmap shows global gene expression profiling of CRC tissues versus nontumor tissues (fold change > 2, $P < 0.01$, FDR < 0.01). Red and green colors represent up-regulation and down-regulation, respectively.
