# Supplementary material for: Twa1/Gid8 is a β-catenin nuclear retention factor in Wnt signaling and colorectal tumorigenesis
Source: Cell Res. 2017 Aug 22;27(12):1422–40. doi: 10.1038/cr.2017.107 (PMC5717399; doi:10.1038/cr.2017.107)
Supplement: Supplementary information, Figure S2 — Twa1 is selectively involved in canonical Wnt signaling. [file cr2017107x2.pdf]

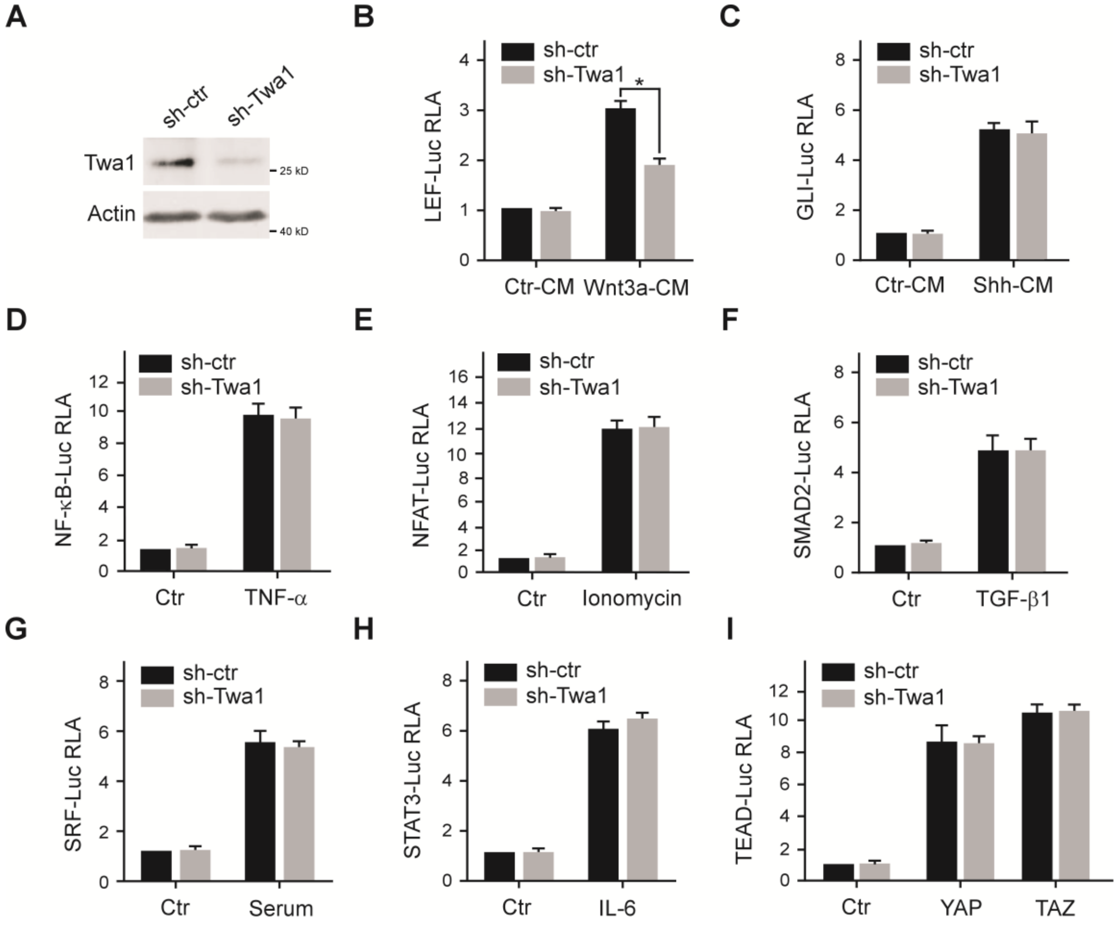
A
sh-ctr
sh-Twa1
Twa1
25 kD
Actin
40 kD
B
sh-ctr
sh-Twa1
*
LEF-Luc RLA
Ctr-CM
Wnt3a-CM
C
sh-ctr
sh-Twa1
GLI-Luc RLA
Ctr-CM
Shh-CM
D
sh-ctr
sh-Twa1
NF-κB-Luc RLA
Ctr
TNF-α
E
sh-ctr
sh-Twa1
NFAT-Luc RLA
Ctr
Ionomycin
F
sh-ctr
sh-Twa1
SMAD2-Luc RLA
Ctr
TGF-β1
G
sh-ctr
sh-Twa1
SRF-Luc RLA
Ctr
Serum
H
sh-ctr
sh-Twa1
STAT3-Luc RLA
Ctr
IL-6
I
sh-ctr
sh-Twa1
TEAD-Luc RLA
Ctr
YAP
TAZ

Supplementary information, Figure S2 Twa1 is selectively involved in canonical Wnt signaling. (**A**) Western analysis showing the efficiency of Twa1 knockdown in HEK-293 cells. The cells were treated with lentivirus-based shRNAs targeting Twa1 (sh-Twa1) or control shRNA (sh-ctr) and subjected to immunoblotting. Actin was used as a loading control. (**B-I**) Effects of Twa1 depletion on the indicated pathways determined by dual luciferase reporter assays. HEK-293 cells infected with lentiviruses containing sh-Twa1 or sh-ctr were transfected with the plasmids containing the indicated reporters, and treated with the indicated signaling pathway activators (**B-H**) or co-transfected with the indicated constructs (**I**). Quantitative data are expressed as the mean ± SEM (at least three independent experiments). *$P < 0.05$, Student's *t* test. Ctr, control, GLI, glioma-associated oncogene (Hedgehog signaling); IL-6, interleukin-6; LEF, lymphoid enhancer-binding factor (Wnt/β-catenin signaling); Luc, luciferase; NF-κB, nuclear factor-κB (TNF-α signaling); NFAT, nuclear factor of activated T cells (Calcium signaling); RLA, relative luciferase activity (firefly/renilla luciferase); Shh-CM, sonic hedgehog-conditioned medium; SMAD2, SMAD family member 2 (TGF-β signaling); SRF, serum response factor (MAPK signaling); STAT3, signal transducers and activators of transcription 3 (JAK/STAT signaling); TAZ, transcriptional coactivator with PDZ-binding motif; TEAD, TEA domain (Hippo signaling). TGF-β1, transforming growth factor-β 1; TNF-α, tumor necrosis factor-α; Wnt3a-CM, Wnt3a-conditioned medium; YAP, Yes-associated protein.
