# Supplementary material for: Twa1/Gid8 is a β-catenin nuclear retention factor in Wnt signaling and colorectal tumorigenesis
Source: Cell Res. 2017 Aug 22;27(12):1422–40. doi: 10.1038/cr.2017.107 (PMC5717399; doi:10.1038/cr.2017.107)
Supplement: Supplementary information, Figure S3 — Knockdown of Twa1 reduces the protein levels of endogenous Twa1 in HEK-293 cells. [file cr2017107x3.pdf]

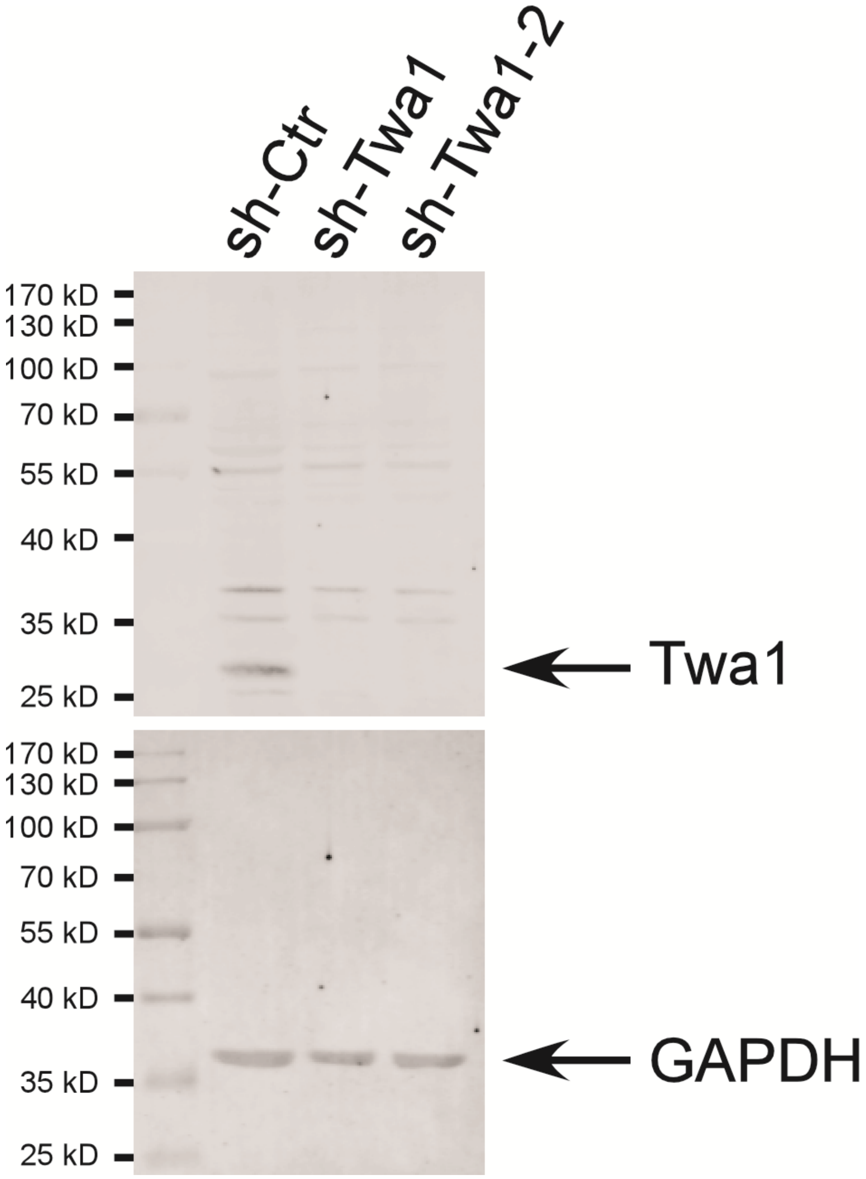
sh-Ctr
sh-Twa1
sh-Twa1-2
170 kD
130 kD
100 kD
70 kD
55 kD
40 kD
35 kD
25 kD
Twa1
170 kD
130 kD
100 kD
70 kD
55 kD
40 kD
35 kD
25 kD
GAPDH

Supplementary information, Figure S3 Knockdown of *Twa1* reduces the protein levels of endogenous Twa1 in HEK-293 cells. The cells transfected with lentivirus-based shRNAs targeting different regions of *Twa1* mRNA (sh-Twa1 and sh-Twa1-2) or control shRNA (sh-ctr) were subjected to western blotting with the indicated antibodies (full immunoblot). GAPDH was used as a loading control.
