# Supplementary material for: Twa1/Gid8 is a β-catenin nuclear retention factor in Wnt signaling and colorectal tumorigenesis
Source: Cell Res. 2017 Aug 22;27(12):1422–40. doi: 10.1038/cr.2017.107 (PMC5717399; doi:10.1038/cr.2017.107)
Supplement: Supplementary information, Figure S4 — Twa1 facilitates β-catenin nuclear accumulation and Wnt target gene expression in RKO cells. [file cr2017107x4.pdf]

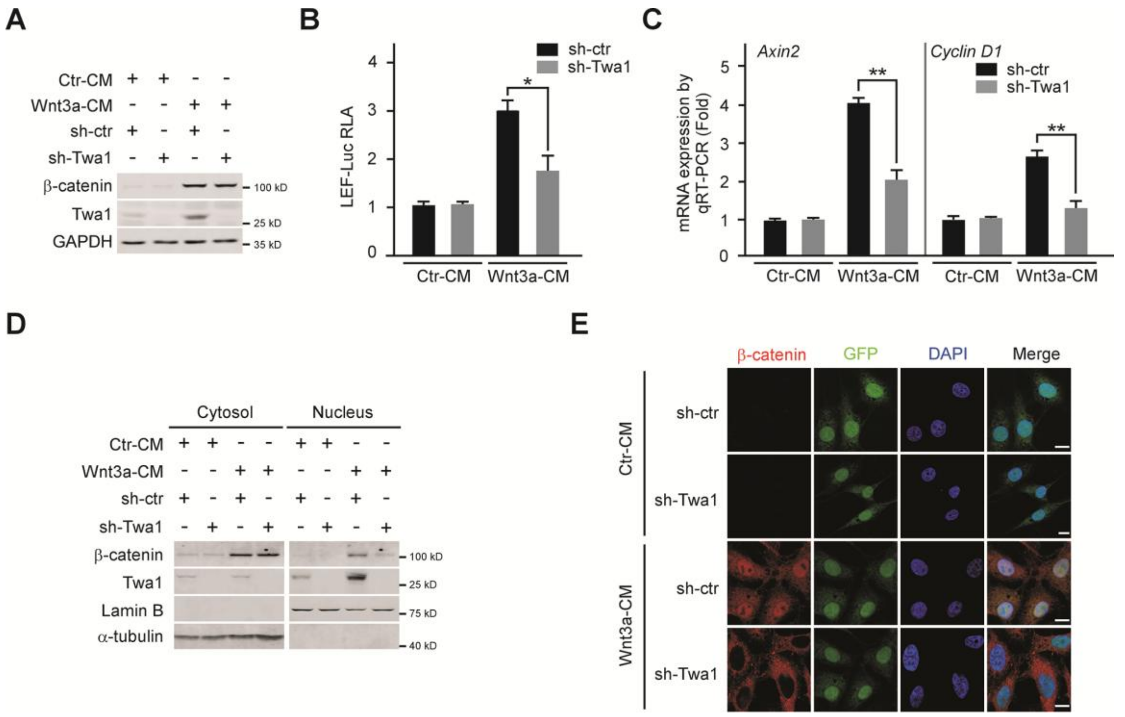
A
Ctr-CM
Wnt3a-CM
sh-ctr
sh-Twa1
β-catenin
Twa1
GAPDH
100 kD
25 kD
35 kD
B
LEF-Luc RLA
sh-ctr
sh-Twa1
Ctr-CM
Wnt3a-CM
C
Axin2
Cyclin D1
mRNA expression by qRT-PCR (Fold)
Ctr-CM
Wnt3a-CM
D
Cytosol
Nucleus
Lamin B
α-tubulin
75 kD
40 kD
E
β-catenin
GFP
DAPI
Merge

Supplementary information, Figure S4 Twa1 facilitates β-catenin nuclear accumulation and Wnt target gene expression in RKO cells. RKO cells infected with lentiviruses containing sh-Twa1 or sh-ctr were treated with Wnt3a-CM or Ctr-CM, and then subjected to the following experiments. (**A**) Western analysis of endogenous β-catenin and Twa1 from RKO cell lysates. GAPDH was used as a loading control. (**B**, **C**) Effects of Twa1 depletion on Wnt-induced luciferase reporter activity **(B)** and Wnt target gene expression by qRT-PCR **(C)**. Quantitative data are shown as the mean ± SEM (at least three independent experiments). *$P < 0.05$ and **$P < 0.01$, Student's *t* test. (**D**) Western blotting of endogenous β-catenin and Twa1 from cytosolic and nuclear fractions of HEK-293 cells. Lamin B and α-tubulin were used as loading controls for nuclear and cytoplasmic fractions, respectively. (**E**) Confocal microscopy showing the nuclear localization of β-catenin. Green signals indicate the cells infected with lentiviruses. DNA was stained with DAPI (blue). Bars, 10 μm.
