# Supplementary material for: Twa1/Gid8 is a β-catenin nuclear retention factor in Wnt signaling and colorectal tumorigenesis
Source: Cell Res. 2017 Aug 22;27(12):1422–40. doi: 10.1038/cr.2017.107 (PMC5717399; doi:10.1038/cr.2017.107)
Supplement: Supplementary information, Figure S5 — Twa1 promotes β-catenin nuclear accumulation and Wnt target gene expression in response to LiCl treatment. [file cr2017107x5.pdf]

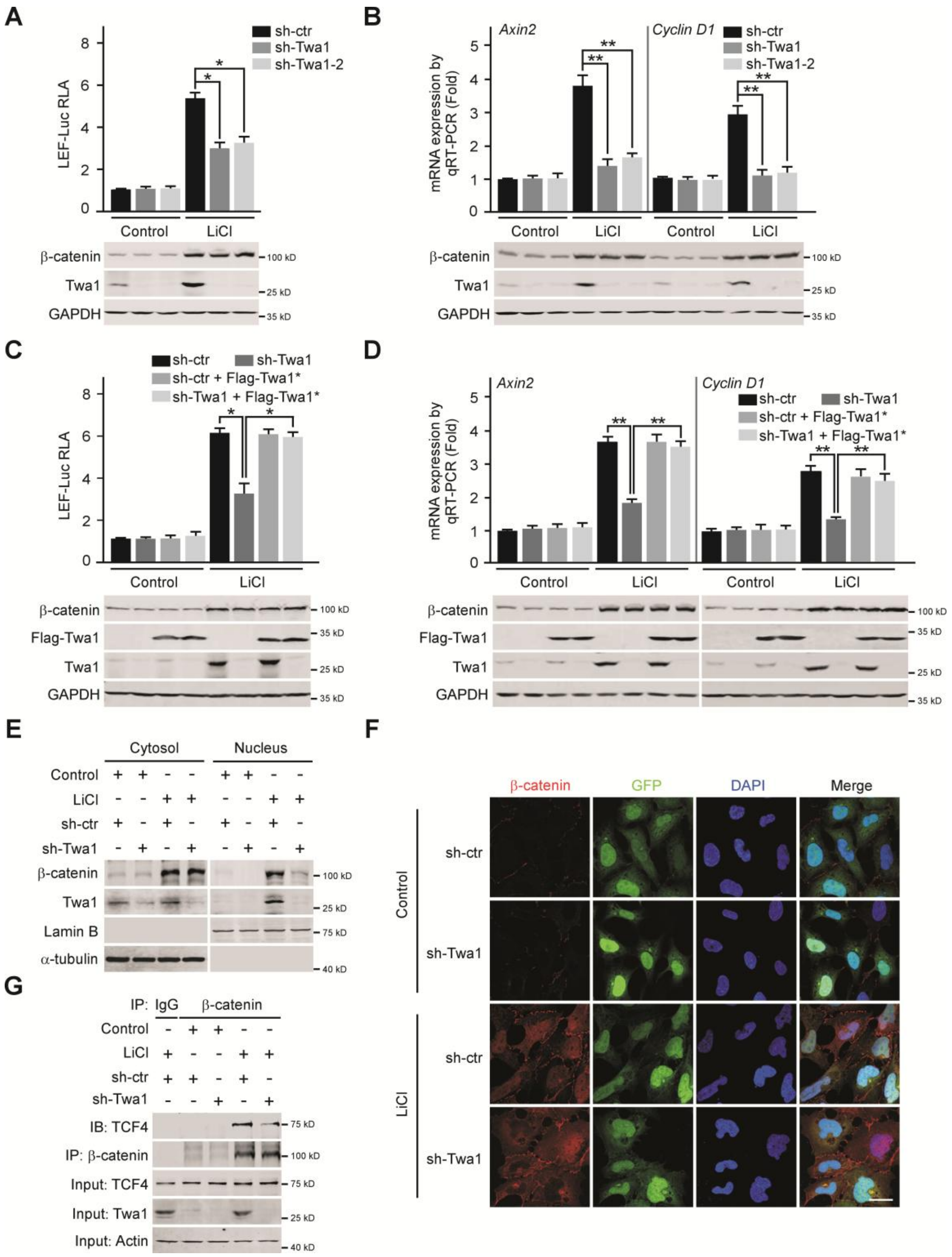
A
LEF-Luc RLA
sh-ctr
sh-Twa1
sh-Twa1-2
Control
LiCl
β-catenin
Twa1
GAPDH
100 kD
25 kD
35 kD
B
Axin2
Cyclin D1
mRNA expression by qRT-PCR (Fold)
C
sh-ctr + Flag-Twa1*
sh-Twa1 + Flag-Twa1*
Flag-Twa1
D
E
Cytosol
Nucleus
Lamin B
α-tubulin
75 kD
40 kD
F
GFP
DAPI
Merge
G
IP: IgG β-catenin
IB: TCF4
IP: β-catenin
Input: TCF4
Input: Twa1
Input: Actin

Supplementary information, Figure S5 Twa1 promotes β-catenin nuclear accumulation and Wnt target gene expression in response to LiCl treatment. HEK-293 cells infected with lentiviruses containing sh-Twa1, sh-Twa1-2 or sh-ctr were transfected with an RNAi-resistant human Twa1 (Twa1*) construct or not. The cells were then treated with LiCl (40 mM) or not, and subjected to the following experiments. (**A**-**D**) Effect of Twa1 depletion on LiCl-induced luciferase reporter activity (**A**, **C**) and Wnt target gene expression by qRT-PCR (**B**, **D**). Quantitative data are shown as the mean ± SEM (at least three independent experiments). $^{*}P < 0.05$ and $^{**}P < 0.01$, Student's *t* test. (**E**) Western blotting of endogenous β-catenin and Twa1 from cytosolic and nuclear fractions of HEK-293 cells. Lamin B and α-tubulin were used as loading controls for nuclear and cytoplasmic fractions, respectively. (**F**) Confocal microscopy showing the nuclear localization of β-catenin. Green signals indicate the cells infected with lentiviruses. DNA was stained with DAPI (blue). Bars, 10 μm. (**G**) Western analysis of the indicated proteins co-immunoprecipitated by anti-β-catenin antibody in HEK-293 cells. IB, immunoblotting; IP, immunoprecipitation.
