# Supplementary material for: Twa1/Gid8 is a β-catenin nuclear retention factor in Wnt signaling and colorectal tumorigenesis
Source: Cell Res. 2017 Aug 22;27(12):1422–40. doi: 10.1038/cr.2017.107 (PMC5717399; doi:10.1038/cr.2017.107)
Supplement: Supplementary information, Figure S6 — Knockout of Twa1 suppresses β-catenin nuclear accumulation and Wnt activation. [file cr2017107x6.pdf]

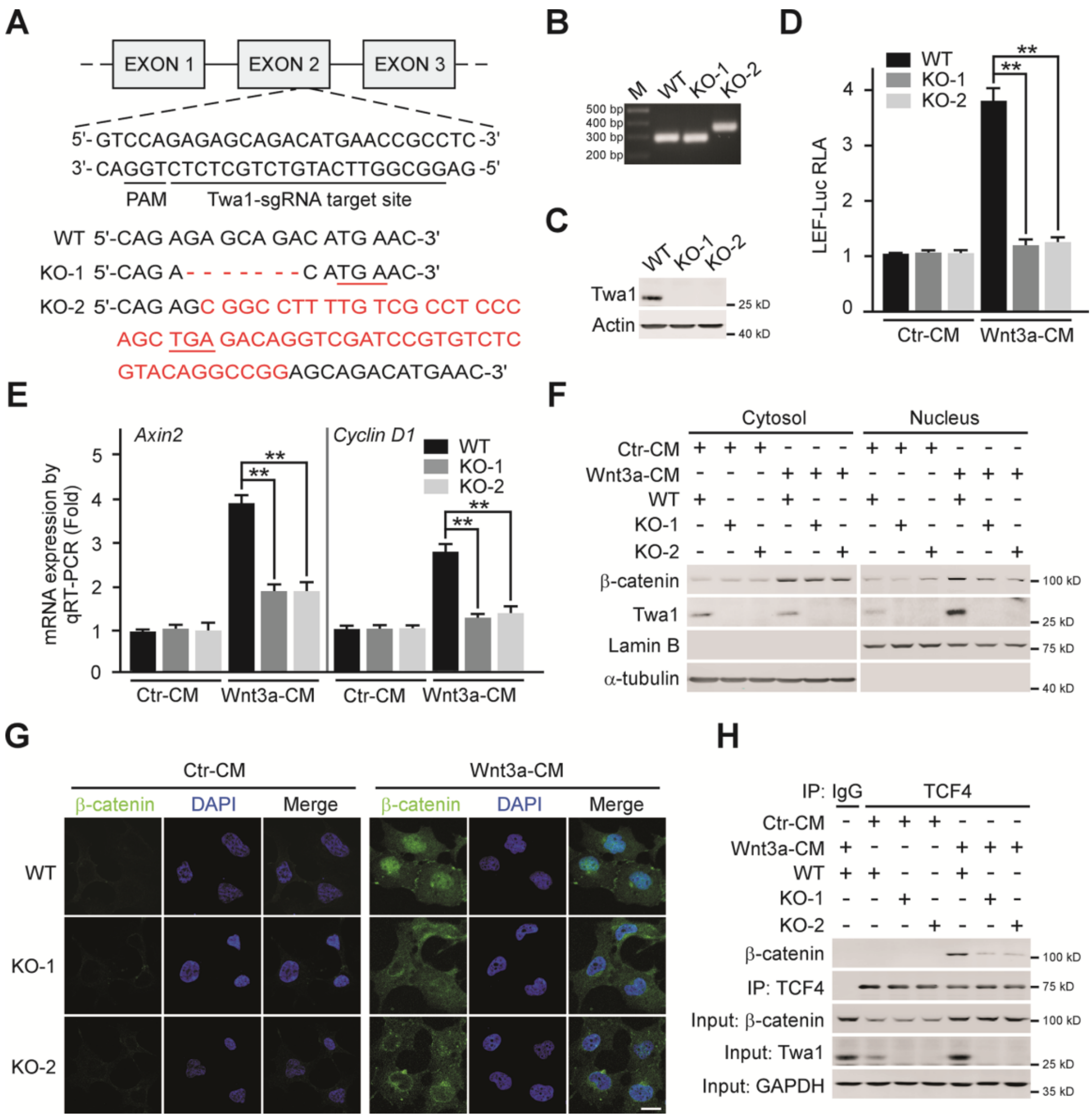
A
EXON 1
EXON 2
EXON 3
5'- GTCCAGAGAGCAGACATGAACCGCCTC -3'
3'- CAGGTCTCTCGTCTGTACTTGGCGGAG -5'
PAM
Twa1-sgRNA target site
WT 5'-CAG AGA GCA GAC ATG AAC-3'
KO-1 5'-CAG A- - - - - - - -C ATG AAC-3'
KO-2 5'-CAG AGC GGC CTT TTG TCG CCT CCC
AGC TGA GACAGGTCGATCCGTGTCTC
GTACAGGCCGGAGCAGACATGAAC-3'
B
M WT KO-1 KO-2
500 bp
400 bp
300 bp
200 bp
C
WT KO-1 KO-2
Twa1
Actin
25 kD
40 kD
D
WT
KO-1
KO-2
LEF-Luc RLA
Ctr-CM
Wnt3a-CM
E
Axin2
Cyclin D1
mRNA expression by qRT-PCR (Fold)
Ctr-CM
Wnt3a-CM
F
Cytosol
Nucleus
Ctr-CM
Wnt3a-CM
WT
KO-1
KO-2
β-catenin
Twa1
Lamin B
α-tubulin
100 kD
25 kD
75 kD
40 kD
G
Ctr-CM
Wnt3a-CM
β-catenin
DAPI
Merge
WT
KO-1
KO-2
H
IP: IgG
TCF4
Ctr-CM
Wnt3a-CM
WT
KO-1
KO-2
β-catenin
IP: TCF4
Input: β-catenin
Input: Twa1
Input: GAPDH
100 kD
75 kD
100 kD
25 kD
35 kD

Supplementary information, Figure S6 Knockout of *Twa1* suppresses β-catenin nuclear accumulation and Wnt activation. (**A**) Diagram of the sgRNA target site and the sequence of indels in the *Twa1* locus in HEK-293 cells generated by the Cas9/sgRNA system. (**B**) Agarose gel electrophoresis of the PCR products from the genomic DNA of wild-type (WT) and *Twa1* knockout HEK-293 cells (KO-1 and KO-2). (**C**) Western analysis of endogenous Twa1 in wild-type and *Twa1* knockout HEK-293 cells. (**D-H**) Wild-type and *Twa1* knockout cells were transfected with the indicated constructs or not, treated with Wnt3a-CM or Ctr-CM, and then subjected to luciferase reporter activity (**D**), Wnt target gene expression (**E**), western blotting (**F**), confocal microscopy (**G**) and co-IP analyses (**H**). DNA was visualized with DAPI (blue). Scale bars, 10 μm. Quantitative data are shown as the mean ± SEM (at least three independent experiments). $^{**}P < 0.01$, Student's *t* test.
