# Supplementary material for: Twa1/Gid8 is a β-catenin nuclear retention factor in Wnt signaling and colorectal tumorigenesis
Source: Cell Res. 2017 Aug 22;27(12):1422–40. doi: 10.1038/cr.2017.107 (PMC5717399; doi:10.1038/cr.2017.107)
Supplement: Supplementary information, Figure S7 — Twa1 binds to β-catenin in vitro and in vivo. [file cr2017107x7.pdf]

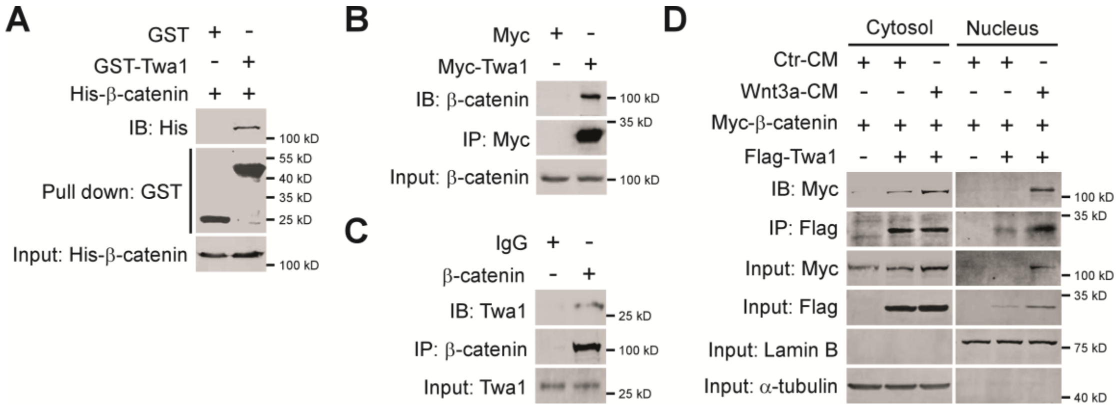

A
GST
GST-Twa1
His-β-catenin
IB: His
100 kD
Pull down: GST
55 kD
40 kD
35 kD
25 kD
Input: His-β-catenin
100 kD
B
Myc
Myc-Twa1
IB: β-catenin
100 kD
IP: Myc
35 kD
Input: β-catenin
100 kD
C
IgG
β-catenin
IB: Twa1
25 kD
IP: β-catenin
100 kD
Input: Twa1
25 kD
D
Cytosol
Nucleus
Ctr-CM
Wnt3a-CM
Myc-β-catenin
Flag-Twa1
IB: Myc
100 kD
IP: Flag
35 kD
Input: Myc
100 kD
Input: Flag
35 kD
Input: Lamin B
75 kD
Input: α-tubulin
40 kD

**Supplementary information, Figure S7** Twa1 binds to β-catenin *in vitro* and *in vivo*. (**A**) GST pull-down analysis of purified His-β-catenin and GST-Twa1 proteins *in vitro*. (**B**, **C**) HEK-293 cells transfected with the indicated plasmids (**B**) or not (**C**) were subjected to co-IP analysis with the indicated antibodies. (**D**) Cytosolic or nuclear extracts from HEK-293 cells transfected with the indicated constructs and treated with Wnt3a-CM or Ctr-CM were subjected to co-IP experiments with anti-Flag antibody. Lamin B and α-tubulin were used as loading controls for nuclear and cytoplasmic fractions, respectively.
