# Supplementary material for: Twa1/Gid8 is a β-catenin nuclear retention factor in Wnt signaling and colorectal tumorigenesis
Source: Cell Res. 2017 Aug 22;27(12):1422–40. doi: 10.1038/cr.2017.107 (PMC5717399; doi:10.1038/cr.2017.107)
Supplement: Supplementary information, Figure S8 — Subcellular localization of endogenous Twa1 in HEK-293 cells. [file cr2017107x8.pdf]

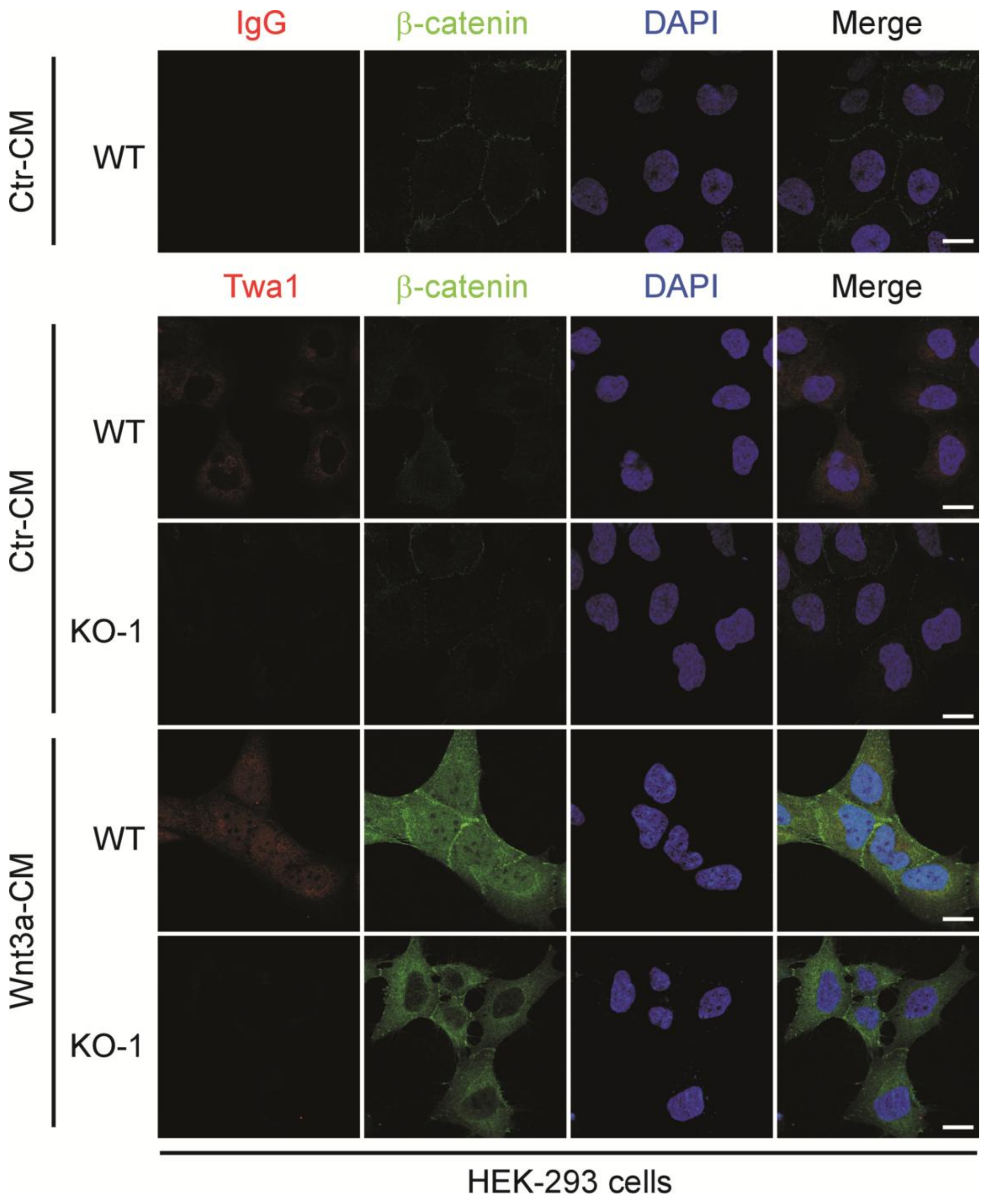

IgG
β-catenin
DAPI
Merge
Ctr-CM
WT
Twa1
β-catenin
DAPI
Merge
Ctr-CM
WT
KO-1
Wnt3a-CM
WT
KO-1
HEK-293 cells

Supplementary information, Figure S8 Subcellular localization of endogenous Twa1 in HEK-293 cells. Wild-type and *Twa1* knockout cells were treated with Wnt3a-CM or Ctr-CM, and then subjected to immunostaining with the indicated antibodies or rabbit IgG. DNA was stained with DAPI (blue). Bars, 10 μm.
