# Supplementary material for: Twa1/Gid8 is a β-catenin nuclear retention factor in Wnt signaling and colorectal tumorigenesis
Source: Cell Res. 2017 Aug 22;27(12):1422–40. doi: 10.1038/cr.2017.107 (PMC5717399; doi:10.1038/cr.2017.107)
Supplement: Supplementary information, Figure S9 — Either knockout or knockdown of APC gene increases Twa1 protein levels and its nuclear accumulation in HEK-293 cells. [file cr2017107x9.pdf]

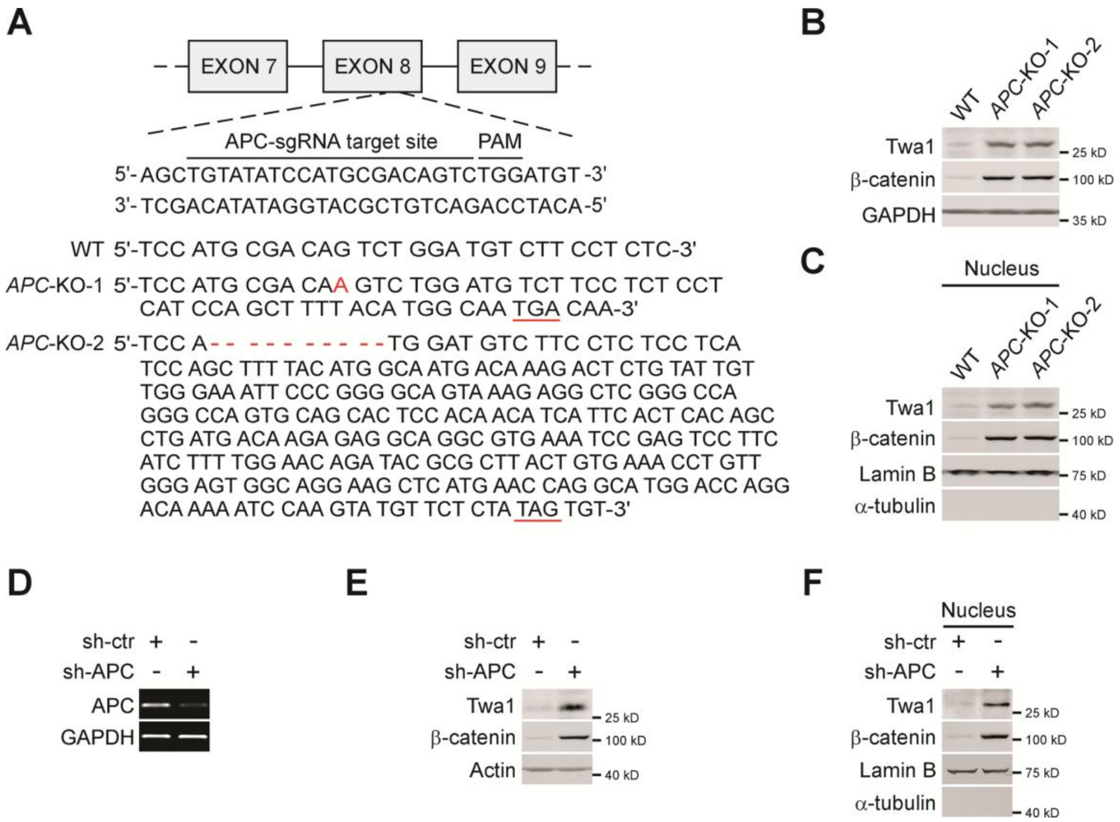
A
EXON 7
EXON 8
EXON 9
APC-sgRNA target site
PAM
5'- AGCTGTATATCCATGCGACAGTCTGGATGT -3'
3'- TCGACATATAGGTACGCTGTCAGACCTACA -5'
WT 5'-TCC ATG CGA CAG TCT GGA TGT CTT CCT CTC-3'
APC-KO-1 5'-TCC ATG CGA CAA GTC TGG ATG TCT TCC TCT CCT
CAT CCA GCT TTT ACA TGG CAA TGA CAA-3'
APC-KO-2 5'-TCC A- - - - - - - - - - -TG GAT GTC TTC CTC TCC TCA
TCC AGC TTT TAC ATG GCA ATG ACA AAG ACT CTG TAT TGT
TGG GAA ATT CCC GGG GCA GTA AAG AGG CTC GGG CCA
GGG CCA GTG CAG CAC TCC ACA ACA TCA TTC ACT CAC AGC
CTG ATG ACA AGA GAG GCA GGC GTG AAA TCC GAG TCC TTC
ATC TTT TGG AAC AGA TAC GCG CTT ACT GTG AAA CCT GTT
GGG AGT GGC AGG AAG CTC ATG AAC CAG GCA TGG ACC AGG
ACA AAA ATC CAA GTA TGT TCT CTA TAG TGT-3'
B
WT
APC-KO-1
APC-KO-2
Twa1
β-catenin
GAPDH
25 kD
100 kD
35 kD
C
Nucleus
WT
APC-KO-1
APC-KO-2
Twa1
β-catenin
Lamin B
α-tubulin
25 kD
100 kD
75 kD
40 kD
D
sh-ctr + -
sh-APC - +
APC
GAPDH
E
sh-ctr + -
sh-APC - +
Twa1
β-catenin
Actin
25 kD
100 kD
40 kD
F
Nucleus
sh-ctr + -
sh-APC - +
Twa1
β-catenin
Lamin B
α-tubulin
25 kD
100 kD
75 kD
40 kD

Supplementary information, Figure S9 Either knockout or knockdown of *APC* gene increases Twa1 protein levels and its nuclear accumulation in HEK-293 cells. (**A**) Diagram of the sgRNA target site and the sequence of indels in the *APC* locus generated by the Cas9/sgRNA system. (**B**, **C**) Cell lysate or nuclear fraction extracted from wild-type and *APC* knockout cells (*APC*-KO-1 and -2) was processed for western blotting with the indicated antibodies. (**D-F**) HEK-293 cells were treated with lentivirus-based shRNAs targeting *APC* (sh-APC) or control shRNA (sh-ctr) and subjected to RT-PCR and Western analyses. RT-PCR showing the efficiency of APC knockdown in HEK-293 cells **(D)**. GAPDH was used as a loading control. Cell lysates or nuclear fractions extracted from HEK-293 cells were processed for western blotting with the indicated antibodies **(E**, **F)**. Lamin B and α-tubulin were used as loading controls for nuclear and cytoplasmic fractions, respectively.
