# Supplementary material for: Twa1/Gid8 is a β-catenin nuclear retention factor in Wnt signaling and colorectal tumorigenesis
Source: Cell Res. 2017 Aug 22;27(12):1422–40. doi: 10.1038/cr.2017.107 (PMC5717399; doi:10.1038/cr.2017.107)
Supplement: Supplementary information, Figure S10 — Characterization of zebrafish homologues of Twa1, Twa1a and Twa1b. [file cr2017107x10.pdf]

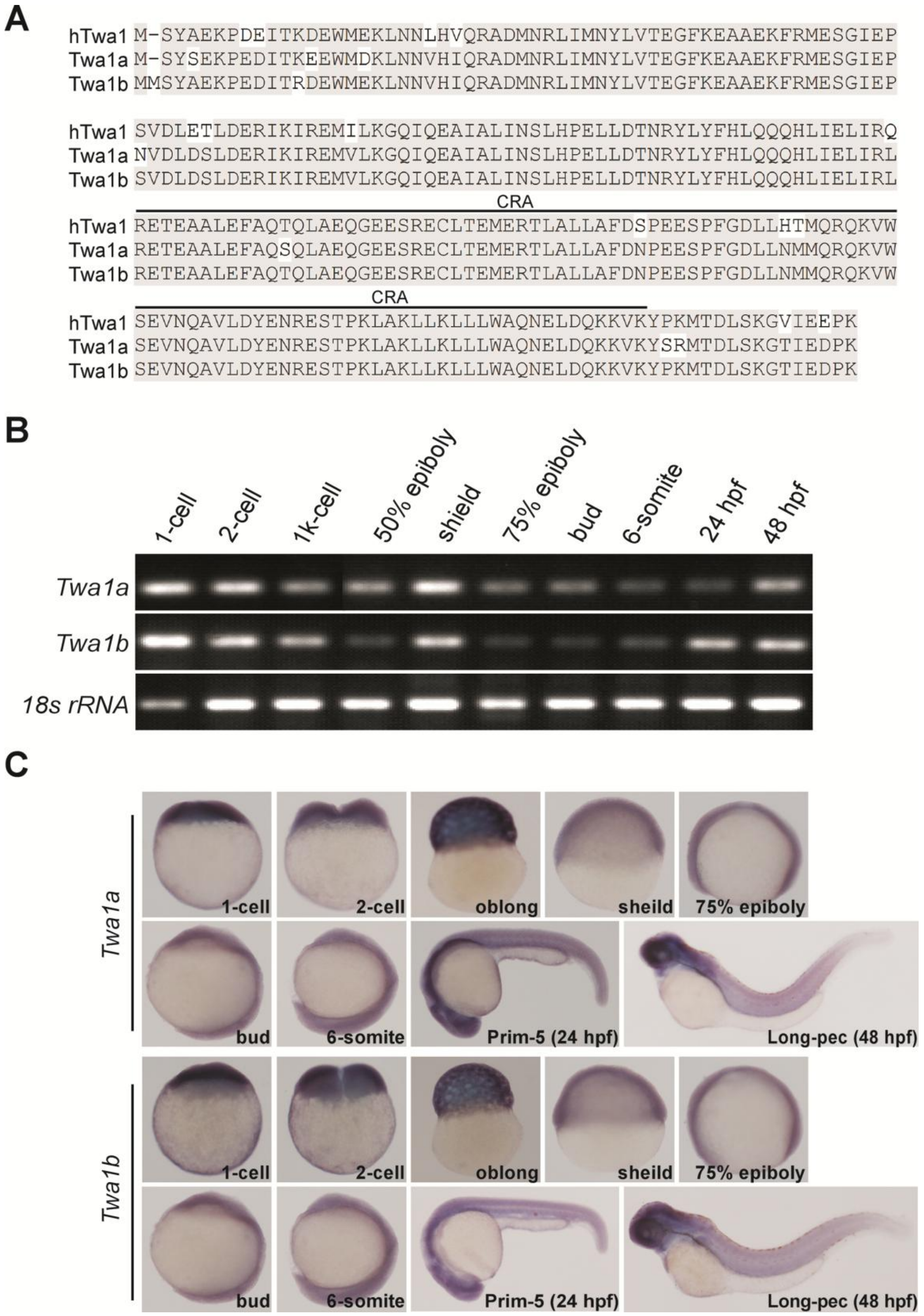
A
hTwa1 M-SYAEKPDEITKDEWMEKLNNLHVQRADMNRLIMNYLVTEGFKEAAEKFRMESGIEP
Twa1a M-SYSEKPEDITKEEWMDKLNNVHIQRADMNRLIMNYLVTEGFKEAAEKFRMESGIEP
Twa1b MMSYAEKPEDITRDEWMEKLNNVHIQRADMNRLIMNYLVTEGFKEAAEKFRMESGIEP
hTwa1 SVDLETLDERIKIREMILKGQIQEAIALINSLHPELLDTNRYLYFHLQQQHLIELIRQ
Twa1a NVDLDSLDERIKIREMVLKGQIQEAIALINSLHPELLDTNRYLYFHLQQQHLIELIRL
Twa1b SVDLDSLDERIKIREMVLKGQIQEAIALINSLHPELLDTNRYLYFHLQQQHLIELIRL
CRA
hTwa1 RETEAALEFAQTQLAEQGEESRECLTEMERTLALLAFDSPEESPFGDLLHTMQRQKVW
Twa1a RETEAALEFAQSQLAEQGEESRECLTEMERTLALLAFDNPEESPFGDLLNMMQRQKVW
Twa1b RETEAALEFAQTQLAEQGEESRECLTEMERTLALLAFDNPEESPFGDLLNMMQRQKVW
CRA
hTwa1 SEVNQAVLDYENRESTPKLAKLLKLLLWAQNELDQKKVKYPKMTDLSKGVIEEPK
Twa1a SEVNQAVLDYENRESTPKLAKLLKLLLWAQNELDQKKVKYSRMTDLSKGTIEDPK
Twa1b SEVNQAVLDYENRESTPKLAKLLKLLLWAQNELDQKKVKYPKMTDLSKGTIEDPK
B
1-cell
2-cell
1k-cell
50% epiboly
shield
75% epiboly
bud
6-somite
24 hpf
48 hpf
Twa1a
Twa1b
18s rRNA
C
Twa1a
Twa1b
1-cell
2-cell
oblong
sheild
75% epiboly
bud
6-somite
Prim-5 (24 hpf)
Long-pec (48 hpf)

Supplementary information, Figure S10 Characterization of zebrafish homologues of *Twa1*, *Twa1a* and *Twa1b*. (**A**) The deduced amino acid sequences of human *Twa1* (*hTwa*1) and zebrafish *Twa1a* (*Twa1a*) and *Twa1b* (*Twa1b*). The CRA domain is underlined. (**B**) RT-PCR was performed on total RNAs isolated from the indicated developmental stages of zebrafish embryos. (**C**) The embryos were fixed at the indicated stages and processed for whole mount *in situ* hybridization with *Twa1a* and *Twa1b* probes. The embryos are shown in lateral view. hpf, hours post-fertilization; Prim-5, Primordia-5; Long-pec, Long-pectoral fin.
