# Supplementary material for: Twa1/Gid8 is a β-catenin nuclear retention factor in Wnt signaling and colorectal tumorigenesis
Source: Cell Res. 2017 Aug 22;27(12):1422–40. doi: 10.1038/cr.2017.107 (PMC5717399; doi:10.1038/cr.2017.107)
Supplement: Supplementary information, Figure S11 — Effectiveness of the morpholinos targeting Twa1a and Twa1b mRNA (Twa1a-MO and Twa1b-MO) in zebrafish embryos. [file cr2017107x11.pdf]

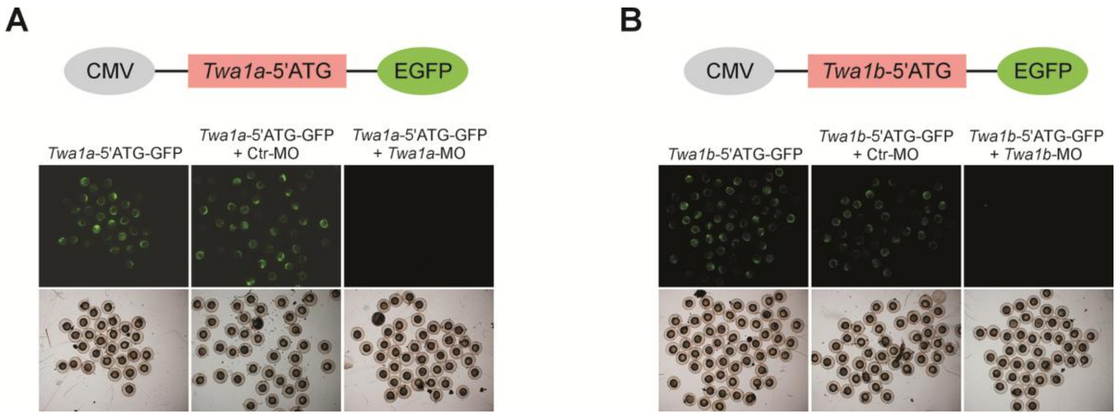
A
CMV
Twa1a-5'ATG
EGFP
Twa1a-5'ATG-GFP
Twa1a-5'ATG-GFP + Ctr-MO
Twa1a-5'ATG-GFP + Twa1a-MO
B
CMV
Twa1b-5'ATG
EGFP
Twa1b-5'ATG-GFP
Twa1b-5'ATG-GFP + Ctr-MO
Twa1b-5'ATG-GFP + Twa1b-MO

Supplementary information, Figure S11 Effectiveness of the morpholinos targeting *Twa1a* and *Twa1b* mRNA (*Twa1a*-MO and *Twa1b*-MO) in zebrafish embryos. (**A**, **B**) The indicated MOs and constructs were injected into embryos at the one-cell stage. GFP signals were observed at 24 hpf. The plasmids contain zebrafish *Twa1a*- or *Twa1b*-5’ATG region carrying the targeting sequence of their corresponding MOs, which are fused with EGFP and driven by a cytomegalovirus (CMV) promoter.
