# Supplementary material for: Twa1/Gid8 is a β-catenin nuclear retention factor in Wnt signaling and colorectal tumorigenesis
Source: Cell Res. 2017 Aug 22;27(12):1422–40. doi: 10.1038/cr.2017.107 (PMC5717399; doi:10.1038/cr.2017.107)
Supplement: Supplementary information, Figure S12 — Depletion of Twa1 inhibits Wnt activation and cell proliferation in SW480 cells. [file cr2017107x12.pdf]

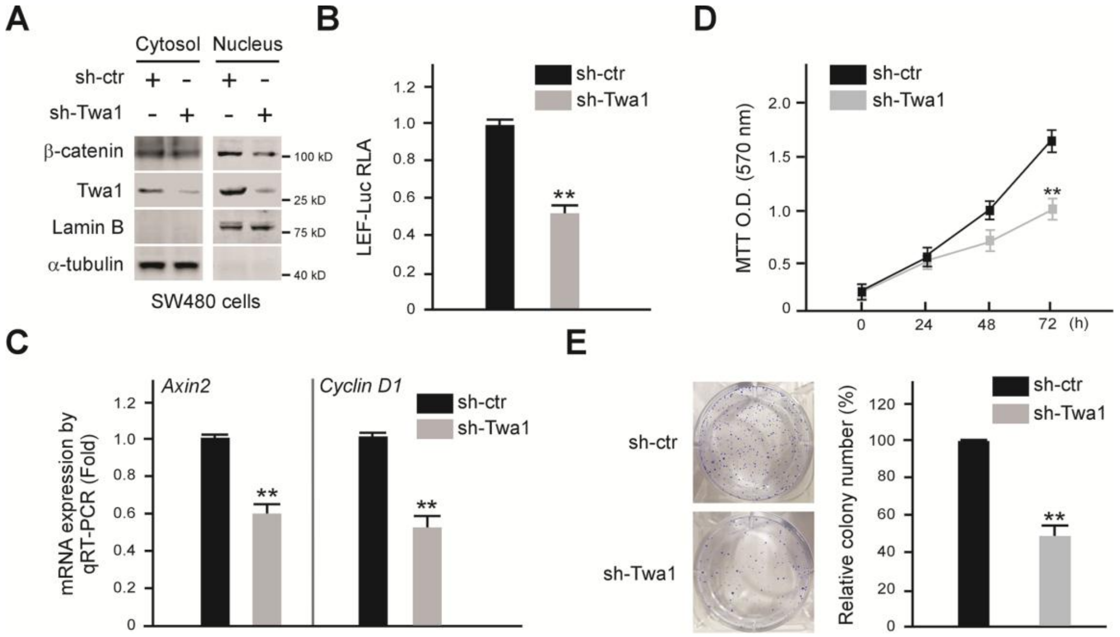
A
Cytosol
Nucleus
sh-ctr
sh-Twa1
β-catenin
Twa1
Lamin B
α-tubulin
100 kD
25 kD
75 kD
40 kD
SW480 cells
B
LEF-Luc RLA
sh-ctr
sh-Twa1
**
D
MTT O.D. (570 nm)
sh-ctr
sh-Twa1
0
24
48
72
(h)
**
C
mRNA expression by qRT-PCR (Fold)
Axin2
Cyclin D1
sh-ctr
sh-Twa1
**
E
sh-ctr
sh-Twa1
Relative colony number (%)
sh-ctr
sh-Twa1
**

Supplementary information, Figure S12 Depletion of Twa1 inhibits Wnt activation and cell proliferation in SW480 cells. (**A-E**) SW480 cells infected with lentiviruses containing sh-Twa1 or sh-ctr were subjected to western blotting (**A**), dual luciferase reporter activity (**B**), Wnt target gene expression (**C**), MTT (**D**) and colony formation analyses (**E**). Lamin B and α-tubulin were used as loading controls for nuclear and cytosolic fractions, respectively. Quantitative data are expressed as the mean ± SEM (at least three independent experiments). ***P* < 0.01, Student's *t* test.
