# Supplementary material for: Twa1/Gid8 is a β-catenin nuclear retention factor in Wnt signaling and colorectal tumorigenesis
Source: Cell Res. 2017 Aug 22;27(12):1422–40. doi: 10.1038/cr.2017.107 (PMC5717399; doi:10.1038/cr.2017.107)
Supplement: Supplementary information, Figure S13 — Twa1 is required for β-catenin nuclear retention in colon cancer cells. [file cr2017107x13.pdf]

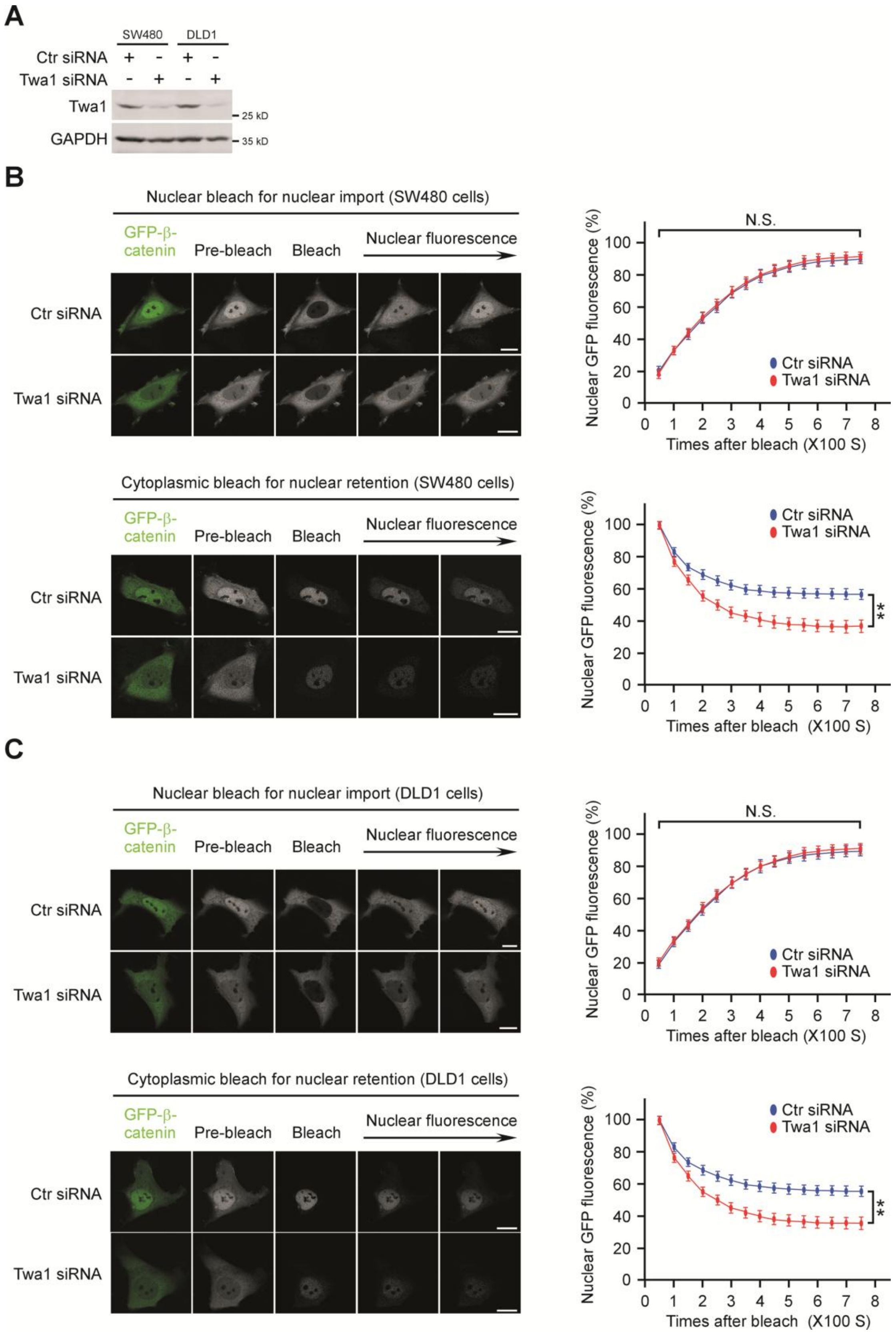

A
SW480
DLD1
Ctr siRNA + - + -
Twa1 siRNA - + - +
Twa1
25 kD
GAPDH
35 kD
B
Nuclear bleach for nuclear import (SW480 cells)
GFP-β-catenin
Pre-bleach
Bleach
Nuclear fluorescence
Ctr siRNA
Twa1 siRNA
N.S.
Nuclear GFP fluorescence (%)
Times after bleach (X100 S)
Cytoplasmic bleach for nuclear retention (SW480 cells)
**
C
Nuclear bleach for nuclear import (DLD1 cells)
Cytoplasmic bleach for nuclear retention (DLD1 cells)

Supplementary information, Figure S13 Twa1 is required for β-catenin nuclear retention in colon cancer cells. (**A**) Western analysis showing the efficiency of Twa1 knockdown in SW480 and DLD1 cells. The cells transfected with Twa1 siRNA or control siRNA (ctr siRNA) were subjected to immunoblotting with the indicated antibodies. GAPDH was used as a loading control. (**B**, **C**) SW480 cells **(B)** or DLD1 cells **(C)** transfected with GFP-β-catenin and the indicated siRNAs were subjected to FRAP analysis. The intensity of nuclear fluorescence was monitored for ~ 750 s and plotted against time. Scale bars, 10 μm. Quantitative data are presented as the mean ± SEM (at least three independent experiments). N.S., not significant. $^{**}P < 0.01$, Student's *t* test.
