# Supplementary material for: Twa1/Gid8 is a β-catenin nuclear retention factor in Wnt signaling and colorectal tumorigenesis
Source: Cell Res. 2017 Aug 22;27(12):1422–40. doi: 10.1038/cr.2017.107 (PMC5717399; doi:10.1038/cr.2017.107)
Supplement: Supplementary information, Figure S14 — Relationship between nuclear Twa1 levels and clinicopathologic features in CRC patients. [file cr2017107x14.pdf]

Relationship between nuclear Twa1 levels and clinicopathologic features in CRC patients

	Nuclear Twa1 expression			
Clinical characteristic	Upregulation	Downregulation	χ^2	*P*
Sex				
Male	40	22	0.796	0.372
Female	32	12		
Age (y)				
< 60	23	11	0.002	0.967
≥ 60	49	23		
Differentiation grade				
Well	14	2	3.348	0.188
Moderate	48	26		
Poor	10	6		
Infiltration depth				
T1	1	8	15.253	0.002
T2	9	5		
T3	20	6		
T4	42	15		
Lymph node metastasis				
N0	34	25	7.559	0.023
N1	26	8		
N2	12	1		
Distant metastasis				
M0	71	34	0.477	0.490
M1	1	0		
Clinical stage				
I	8	10	6.526	0.089
II	25	12		
III	38	12		
IV	1	0		

Note: The statistical significance was determined by χ^2 test. $P < 0.05$ is considered significant. Differentiation grade, infiltration depth, lymph node metastasis, distant metastasis and clinical stage (TNM) were independently evaluated by at least two professional pathologists.

Supplementary information, Figure S14 Relationship between nuclear Twa1 levels and clinicopathologic features in CRC patients.
